# Supplementary material for: Pike: OTU-Level Analysis for Oxford Nanopore Amplicon Metagenomics
Source: Int J Mol Sci. 2025 Apr 28;26(9):4168. doi: 10.3390/ijms26094168 (PMC12071631; doi:10.3390/ijms26094168)
Supplement: Supplementary file 1 [file ijms-26-04168-s001.zip › Supplementary_NEW/PIKE_Supplementary_2.pdf]

Supplementary 2. Benchmarking

In the course of validating our tool, we sought to address several primary inquiry points. Our objective was to identify the number of reads at which all taxa in a specific microbial community were captured and to ascertain the stability of the representation of each organism in the composition. Additionally, we aimed to examine how clustering and the ultimate result of data processing vary with different k-measure sizes.

In addition to validating the algorithm itself and its key aspects, a comparison was also performed with other popular alternatives, including epi2me-labs/wf-metagenomics (with kraken2 and minimap2) and NanoCLUST.

1. Time and memory validation

Supplementary 2 Table.1 Time and memory usages for different reads subsets (for this benchmarking, Snakemake was used). Pike was run in single mode with primer trimming with one thread.

Number of reads	Time, h:m:s	RAM (RSS)
1000	0:03:33	1633.47
5000	0:06:21	2618.5
10000	0:17:00	3759.39
30000	0:27:33	6114.73
50000	0:46:11	10480.64
100000	1:55:53	21504.54

2. Rarefaction curves

The Pike algorithm includes two modes of data analysis: single, in which clustering and consensus building are performed independently for each sample, and pool, in which clustering and consensus construction are carried out jointly for all samples, followed by pulling apart the OTU coverage across samples. Each mode has its own specifics, so we independently benchmarked each of the modes.

First, we aimed to assess data sufficiency using rarefaction curves for different taxonomy levels. Rarefaction was conducted by limiting the maximum number of reads going to Pike clustering using the usereads attribute. In Supplementary 2 Fig.1 and Supplementary 2 Fig.2,

rarefaction was carried out once for each sample, and then the average trend was constructed for all technical replicates from one group of samples.

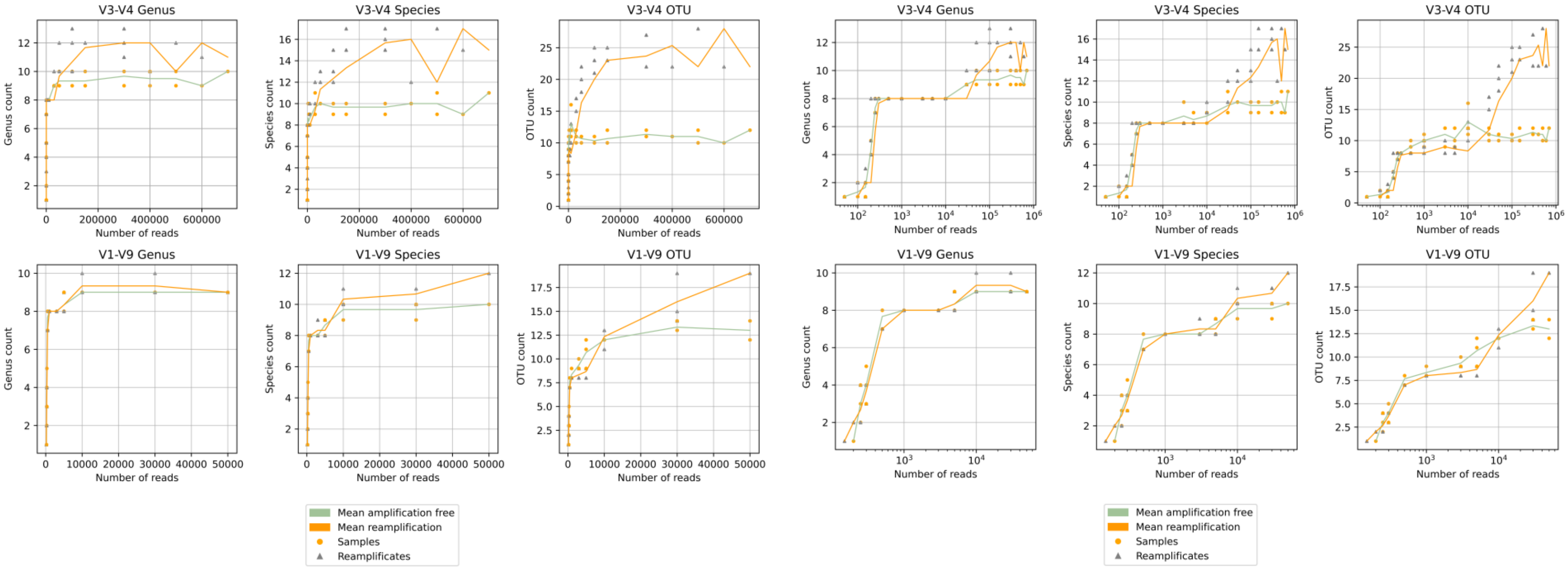

Supplementary 2 Fig.1 Rarefaction curves for 16S mock community data (left - original, right - log scale).

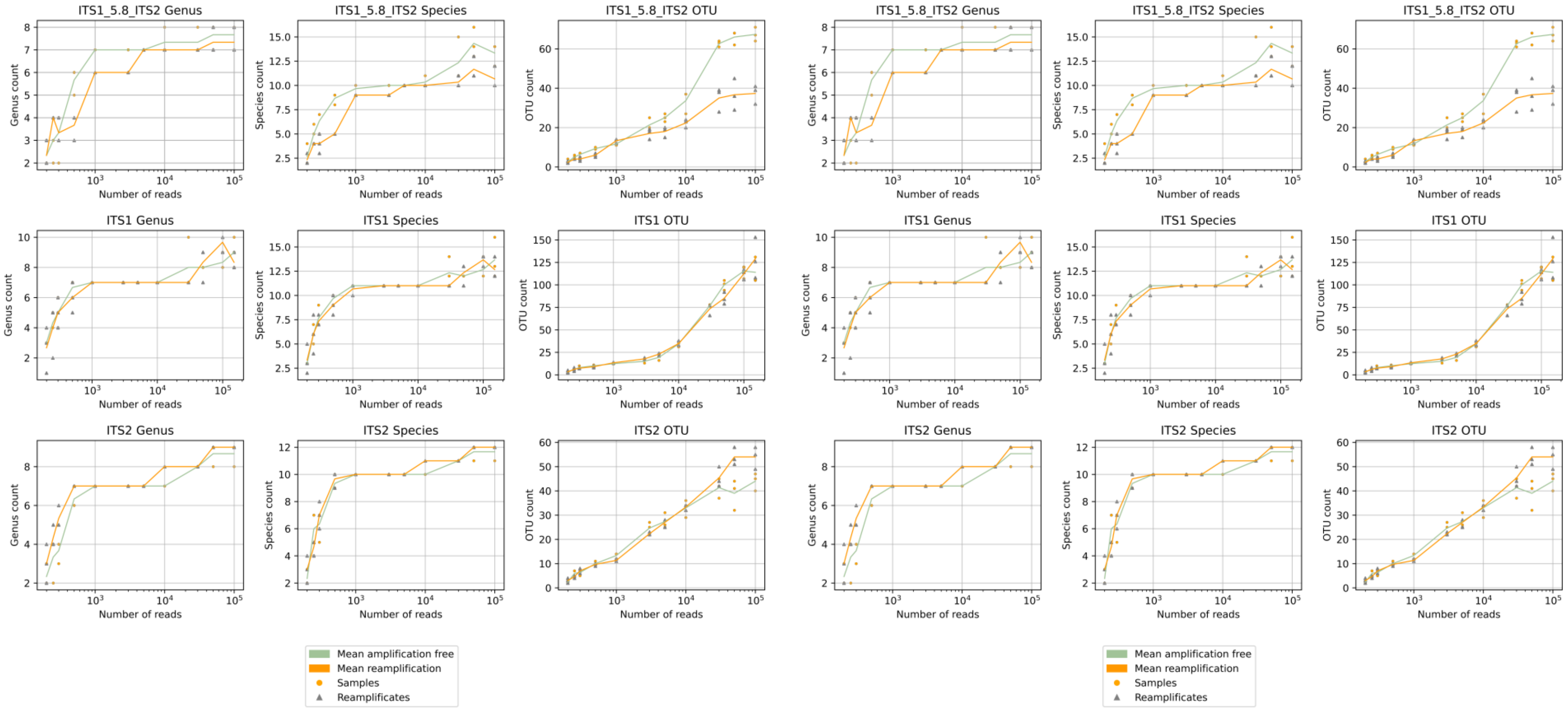

Supplementary 2 Fig.2 Rarefaction curves for ITSs mock community data (left - original, right - log scale).

The results of the analysis indicated that for bacterial communities with a read count of 1000, all eight expected bacterial species were identified. Furthermore, all eight bacterial species were identified, irrespective of the sample group (reamplification free and reamplification groups initially have a similar trend). Next, contamination and an enhancement in the quality of the OTU consensus themselves were documented. Therefore, for the bacterial mock community, starting from 10000 reads, contamination in the form of *Staphylococcus epidermidis* was clearly detected in all samples. The abundance trends of different groups show significant divergence in the case of fungal compositions. Therefore, for the reamplification-free group, all 10 expected fungal species were identified at 3000 reads for ITS1-5.8S-ITS2, at 1000 for ITS1, and at 1000 for ITS2. In the case of reamplification of a group of samples, it was observed that for long amplicons ITS1-5.8S-ITS2, all species were identified at 5000 reads, while for short amplicons (ITS1 and ITS2), all 10 species of fungi were also at 1000 reads. The use of more than 5,000 reads revealed taxonomic inconsistencies in the classification of poorly assembled OTUs, such as s__Rhodotorula_sp and s__Fungi_sp. In practice, such results should be filtered based on the prevalence of taxa in the samples. Additionally, it was important to note that the misclassification events were generally stochastic and were not reproducible between repeats.

The pool mode results were somewhat more complex to interpret (Supplementary 3, Figures 5-6) due to the relatively large number of sequences derived from small fractions of low-quality reads. However, an evaluation of the bar charts obtained from the rarefaction results in Supplementary 3 Fig. 10 - Fig. 12 reveals a notable correlation between the results for the expected taxa and those obtained for single mode. For instance, for 1000 reads, all expected taxa for all mixtures were observed.

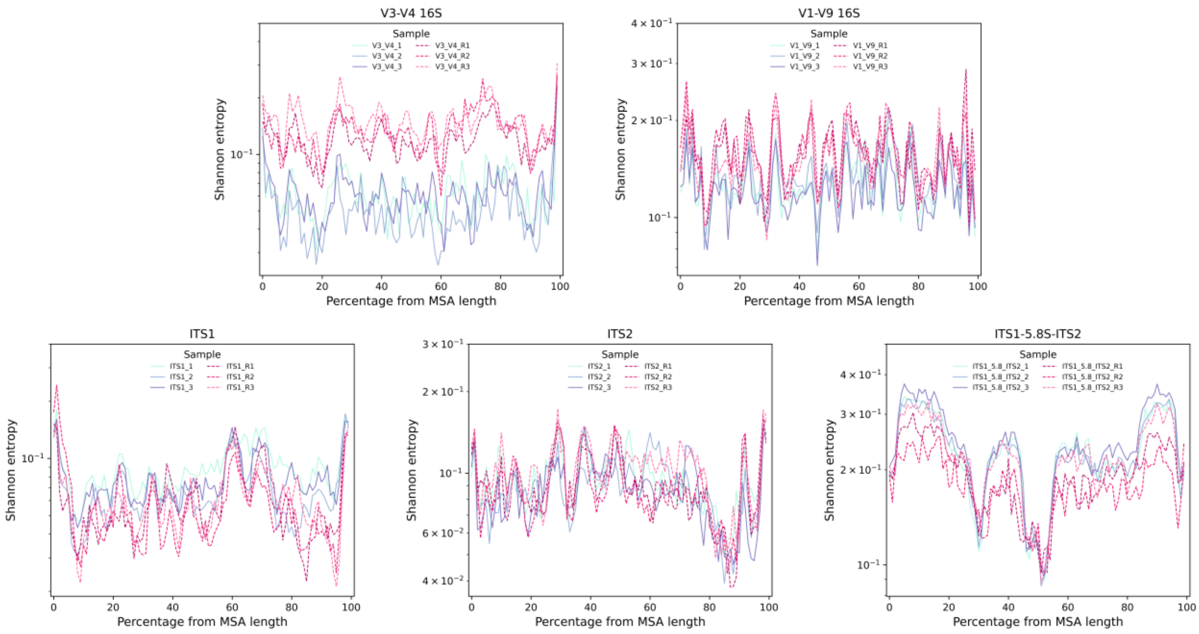

Supplementary 2 Fig.4 Positional change in Shannon information entropy value over multiple alignment for each cluster obtained by Pike (single mode).

Furthermore, it was notable that there was a considerable difference between the mean rarefaction curves for the two groups, with the largest discrepancy observed in samples V1-V9. It was hypothesized that these may be errors introduced during the additional amplification procedure. To test this hypothesis, we calculated the Shannon information entropy values for each position in the "net" reads multiple alignment within each cluster. This approach allowed us to assess the diversity within each position in the multiple alignment (Supplementary 2, Figure 4). It was notable that the entropy values for samples that have undergone an additional amplification stage exhibit higher information entropy values, which likely indicate a greater accumulation of errors during amplification. Additionally, it was observed that the gap between the Shannon information entropy values for samples from the reamplification-free and reamplification groups for amplicons V1-V9 was greater than the difference observed for amplicons V3-V4.

For fungal mock communities, the opposite situation was observed when the curve of the reamplified samples was lower than that of the original samples. As previously indicated in the main text of this study, the fungal compositions were significantly distorted during the reamplification process, so there could be several reasons for the observed phenomenon.

3. Amplification bias

The kinetics of the amplification reaction process can be influenced by a whole host of factors. We assumed that the length of the amplified fragment and the GC composition would influence the amplification to a greater extent. In order to test this, we decided to evaluate the relationship between the median length of the collected OTU for each species and its GC composition.

The most notable cases of the impact of amplification on microbial composition were observed in samples with long amplicons. Furthermore, this phenomenon was most evident in the case of long ITS amplicons (ITS1-5.8S-ITS2). It was postulated that this phenomenon was attributable to the length of the amplicon and its GC composition. Next, we calculated the difference in relative representation proportions for each pair of samples.

We thus proceeded to analyze the changes in microbial composition that occurred during reamplification, using data from long amplicons obtained through Pike's single mode processing. To minimize the impact of technical artifacts resulting from incorrect OTU assembly, only taxa present in all samples were considered. Additionally, bacterial species were considered in this analysis to circumvent taxonomic inconsistencies that emerged during the taxonomic classification process, as detailed in Section 3.2. Fungal species compositions were also examined. The length of each taxon was determined as the median length of all collected OTU sequences belonging to that particular taxon. Similarly, the median proportion of GC content for all collected OTU sequences of a specific taxon was calculated. All results were presented in Fig. 4.

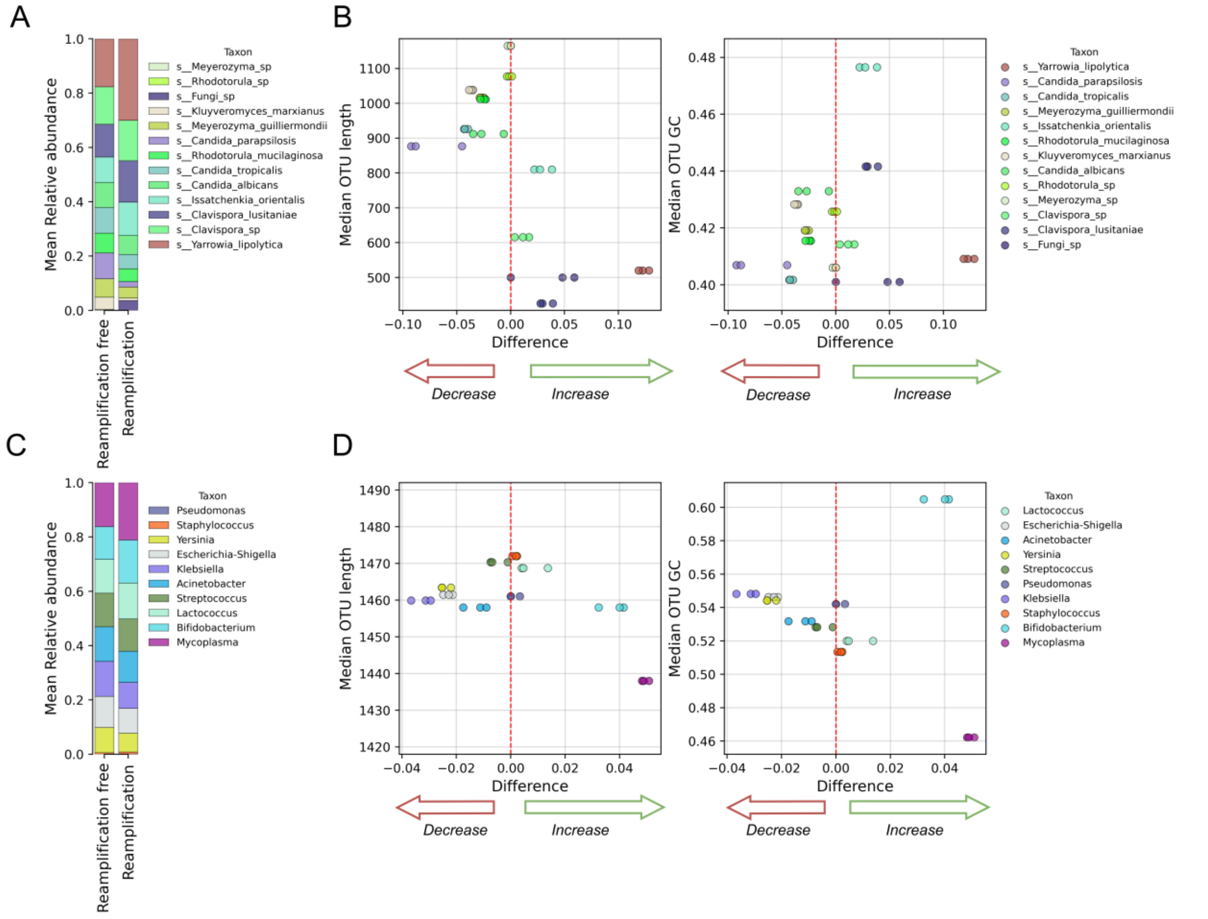

Fig4. Results of assessing the influence of amplicon length and GC composition on the composition. A - mean relative abundance for samples without reamplification and with reamplification for ITS1-5.8S-ITS2; B - graphs of the median OTU length and GC composition versus the difference between pairs before reamplification for ITS1-5.8S-ITS2; C - averaged proportions of relative abundances for samples without reamplification and with reamplification for V1-V9 16S rRNA; D - graphs of median OTU length and GC composition versus the difference between pairs before reamplification for V1-V9 16S rRNA.

As it was expected, the ITS lengths in our mock mixture have greater variability than the one of the V1-V9 region of 16S rRNA. It was therefore evident that pronounced length variability has a considerable impact on the distortion of reamplification outcomes. In the case of bacterial mixtures, the variation in V1-V9 lengths was significantly lower, and no discernible shifts in bacterial composition are observed following reamplification.

It was worth considering in more detail the fungal compositions in which an increase in the relative abundance of *Yarrowia lipolytica* was noted in the samples after reamplification. At the same time, the representation of *Kluyveromyces marxianus* significantly decreases. This can be attributed to the relatively low median length of *Yarrowia lipolytica* (340 bp) and the comparatively high median length of *Kluyveromyces marxianus* (1100 bp). Distributions of median OTU lengths with a difference value less than 0 and distributions of median OTU lengths with a difference value greater than 0 have statistically significant differences (p-value

< 0.05 via Mann-Whitney U test). The results obtained are in agreement with previously published work. In particular, in [1] the authors write that the amplification of shorter fragments has a higher amplification efficiency compared to longer fragments, which we also observe in our results.

It should be noted that there were likely numerous additional factors contributing to the observed shifts. However, within the context of this study, we have focused on the most prominent factors associated with the amplification bias.

4. Restoring a microbial composition

The stability of microbial compositions was evaluated through a visual assessment of the constructed bar charts, with an illustrative example provided in the figure Supplementary 2 Fig.5. Furthermore, comparable images were obtained for all microbial mixtures and for both single and pool modes.

It was observed that for all mixtures, results were more similar when starting from 1000 reads at the genus and species level. In contrast, at the OTU level, convergence was observed to be weak. It was important, however, that the major OTU sequences obtained exhibit relatively weak differences in bacterial communities. In the case of fungal mixtures, however, we hypothesize that there may be heterogeneity in the source population within individual fungal isolates. In other words, we think that our fungal compositions do not contain one correct ITS variant for each taxon.

For pool mode, we would also like to emphasize the problems associated with clustering of similar sequences. Despite the fact that in general, in pool mode, we see convergence of the obtained OTU variants for very similar sequences in very different ratios, it may not be possible to obtain unique clusters. However, the polished mode was more sensitive and was more likely to detect low-represented species.

It was important to note that each approach has its own set of advantages and disadvantages. Pool mode, however, was a comprehensive solution that addresses the heterogeneity of individual taxa. To gain a deeper understanding of the diversity within species, a more detailed analysis of OTUs was necessary.

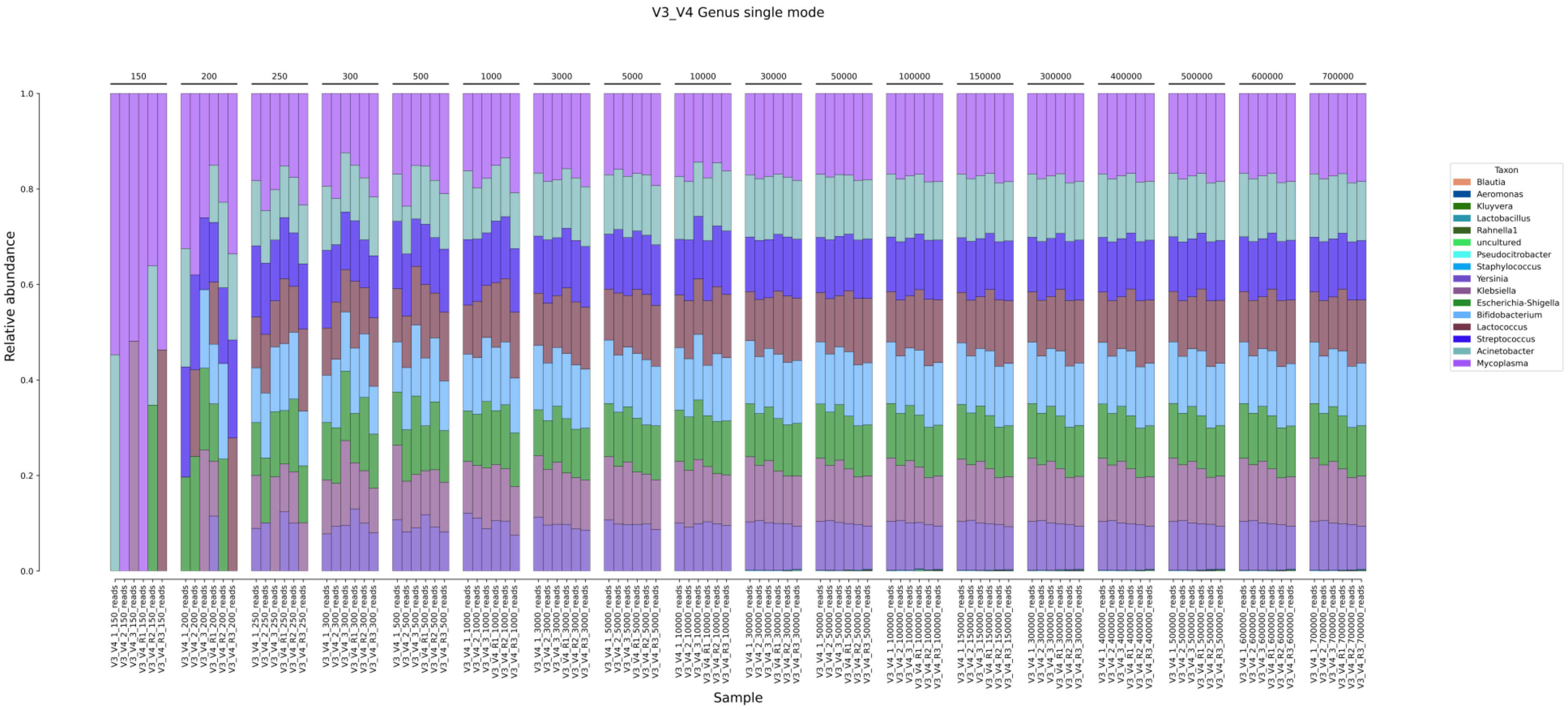

Supplementary 2 Fig.5 Rarefaction results for individual read arrays for V3-V4 (Genus level, single mode).

Furthermore, to provide a more visualization, line plots were created to illustrate changes in taxa representation. Each line plot corresponds to a specific organism (see Supplementary 3, Figures 19–22).

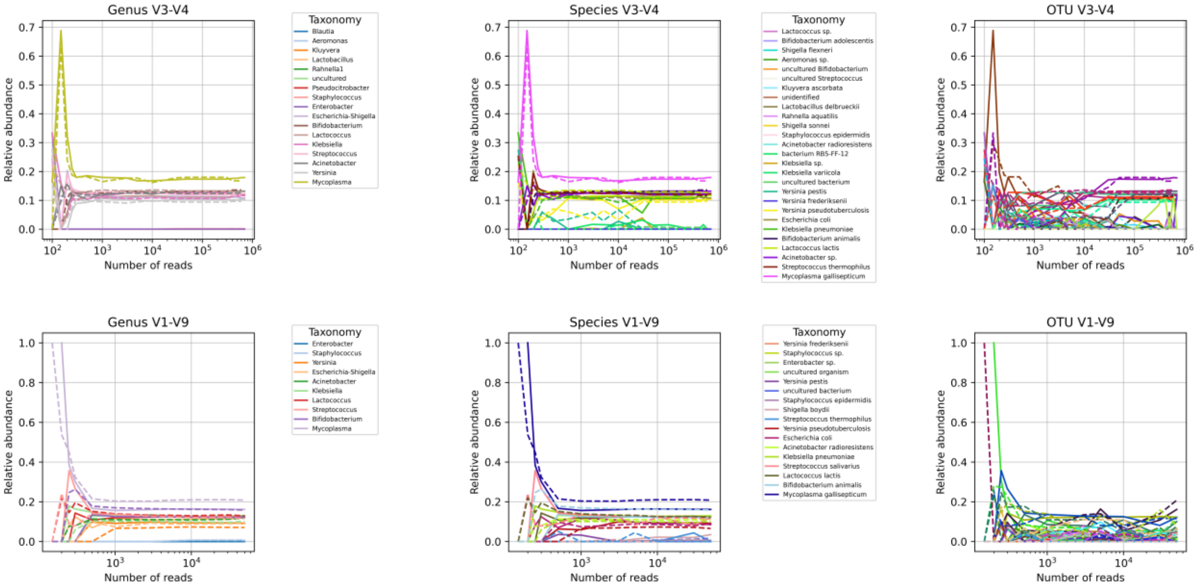

Supplementary 2 Fig.6 Change in relative representation as a function of the number of input reads (log scale). Bacterial community, **single mode.**

5. K-mer size validation

In addition to the previously mentioned objectives, the validation process was designed to assess how the microbial composition changes depending on the selected k-mer size. To evaluate this, we plotted changes in relative representation values as a function of the size of the selected k-mer. No discernible alterations in the range of relative representation were evident, even with the minimum k-mer size. However, in practice, it appeared that the optimal clustering was achieved with a k-mer size of 6. However, for expeditious calculation and the attainment of an approximate result, it was possible to use a relatively small k-mer size, such as k = 3.

We constructed all images similarly to the previous test with thinning and shifted them to Supplementary 3 Fig.13 - Fig.18 and Supplementary 3 Fig.23 - Fig.24. For some more objective criteria of clustering quality, we used the silhouette score and revealed that the highest performance was achieved at k = 5.

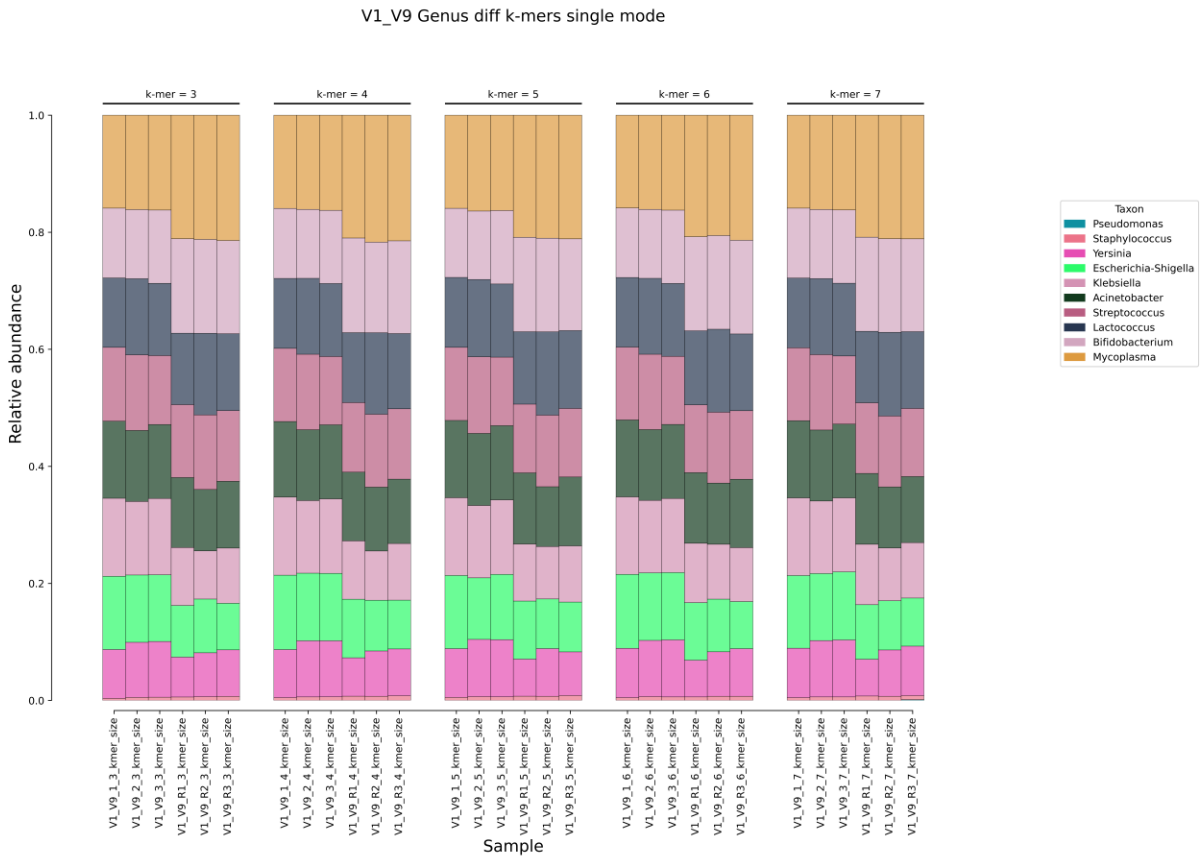

Supplementary 2 Fig.7 Pike's results for V1-V9 with different K-measure sizes (Genus level, **single mode**).

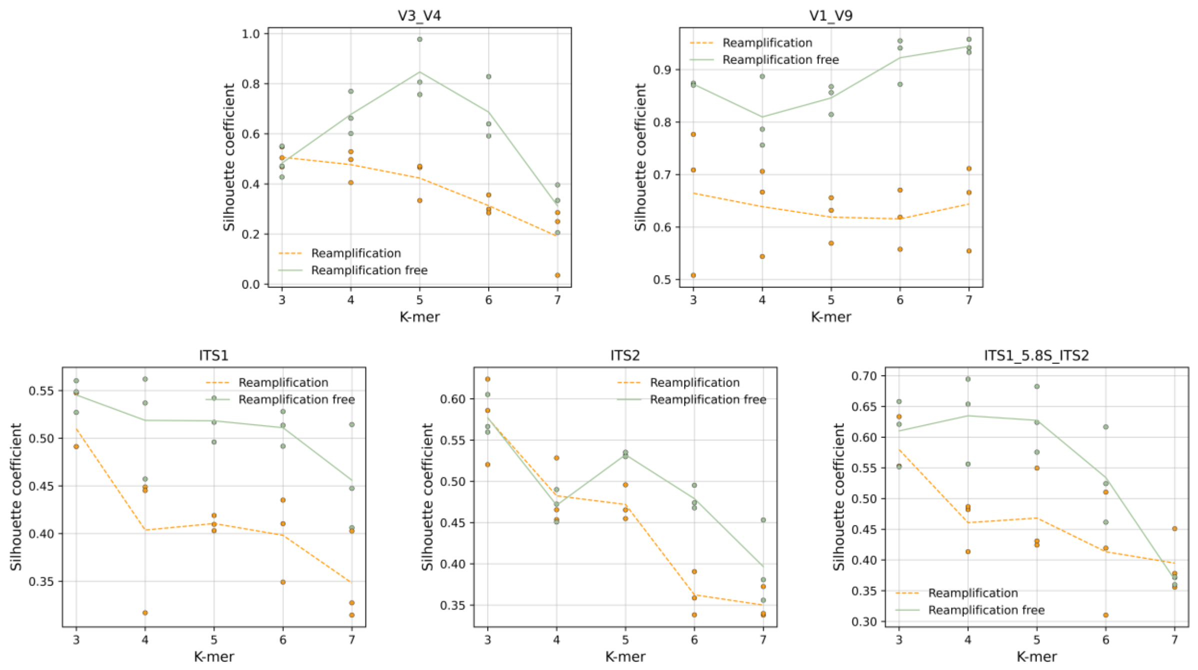

Supplementary 2 Fig.8 Change of Silhouette score value depending on k-measure size (**single mode**).

Next, for single mode, we elected to examine the parametric UMAP two-dimensional decomposition images, wherein we discerned markedly better clustering for bacterial mixture samples.

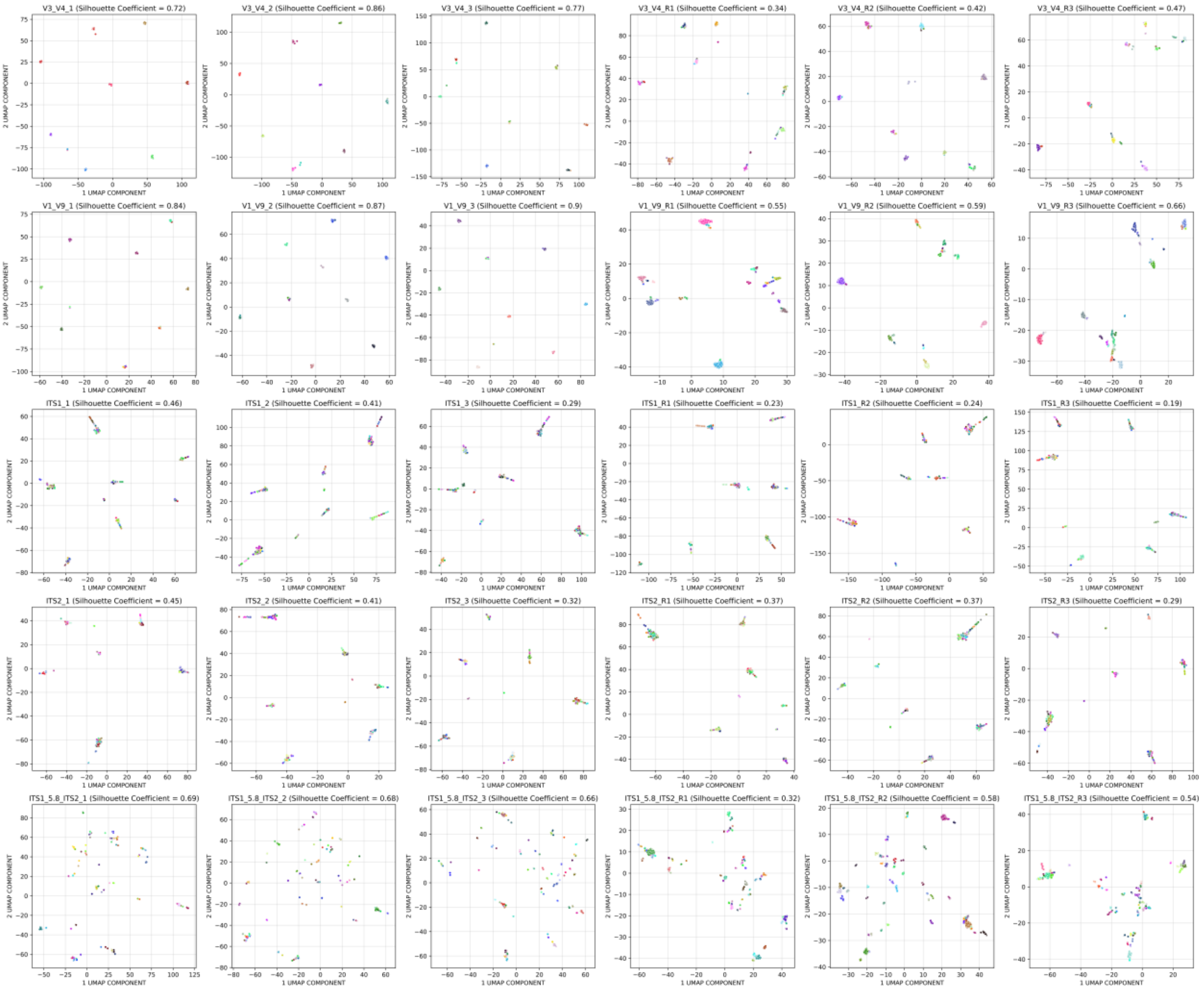

Supplementary 2 Fig.9 Decomposition results using UMAP and clustering using HDBSCAN (**single mode**).

Among interesting observations, we also noted that the silhouette score values in the reamplification free group were larger than those in the reamplification group. Additionally, the silhouette score values for long type amplicons (ITS1-5.8S-ITS2 and V1-V9 16S rRNA) were generally higher than those for short type amplicons.

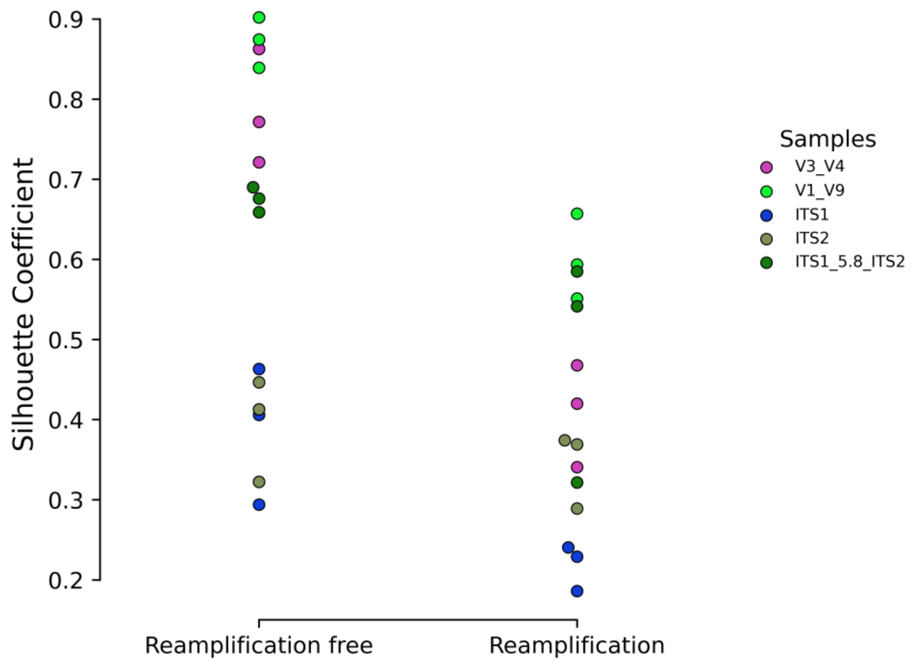

Supplementary 2 Fig.10 Distributions of Silhouette score values for the reamplification free and reamplification groups (**single mode**).

6. Comparison with alternative software

In addition to the tool presented in this work, a number of solutions for analyzing amplicon sequencing data are currently available. In addition to the tool described in this work, two approaches to analyzing this type of data are currently available.

The wf-metagenomics software package included in the epi2me-labs package uses kraken2 or mapping with minimap2. This precludes de novo OTU analysis and restricts microbial community exploration to existing databases. In addition to wf-metagenomics, there is NanoCLUST, which is an effective tool for analyzing microbial communities at the species level. However, the software has a technical limitation regarding the minimum length of the analyzed amplicons, which precludes the assessment of microbial composition obtained using short amplicons such as V3-V4 16S rRNA.

In the case of wf-metagenomics coupled with kraken2, the PlusPF-8 database was employed, whereas for wf-metagenomics in conjunction with minimap2, the NCBI_16s_18s_28s_ITS database was utilized. In the case of NanoCLUST, the default database used for bacterial identification was employed, while the fungal taxonomy was identified using the get_taxonomy.py (with UNITE) function, which is used for Pike. This approach was taken to circumvent the necessity of creating a special database, and it was anticipated that it would not impact the identification of taxa or distort the community composition. The objective was to determine the taxonomy for each collected OTU.

The results of processing the array with all of the presented tools are presented in Supplementary 2 Fig.11 - Fig.15. To minimize visual noise in the images, the legends have

been removed. However, it is evident that wf-metagenomics exhibits a somewhat distorted microbial composition, a phenomenon that is particularly pronounced in the context of short amplicons. Additionally, it was observed that wf-metagenomics with kraken2 incorrectly predicted *Y. pseudotuberculosis*, identifying *S. enterica* instead. In the case of the wf-metagenomics results, specific target organisms are also observed among the major organisms, albeit with a slight compositional shift.

The results obtained for NanoCLUST were found to be highly effective for V1-V9. However, the software limitation on the read length of 1000 bp impeded the calculation of results for V3-V4, ITS1, and ITS2. Furthermore, only a portion of the fungi could be identified for ITS1-5.8S-ITS2. Fungi such as *Y. lipolytica*, which have a length of less than 400 bp, were not detected. Additionally, the OTU level of NanoCLUST is slightly inferior to that of Pike in both single and pool modes. Thus, *Clavispora lusitaniae*, *Candida auris and Yarrowia lipolytica* were not detected using NanoCLUST.

The results of this treatment revealed a number of advantages and disadvantages that are characteristic of alternative tools.

Supplementary 2 Table.2 The advantages and disadvantages we highlighted for the alternative tools we noted during the course of our work.

	wf-metagenomics		NanoCLUST
	kraken2	minimap2	
Advantages	• easy to start and easy to configure<br>• does not depend on read length		• performs OTUs assembling<br>• restores microbial composition well
Disadvantages	• errors in taxonomic identification (*Yersinia* → *Salmonella*)<br>• does not perform OTUs assembling<br>• high rate of false positive identification of taxa<br>• restores microbial composition with inaccuracies	• does not perform OTUs assembling<br>• high rate of false positive identification of taxa<br>• restores microbial composition with inaccuracies	• difficult to install<br>• does not work with short reads (length > 1000)

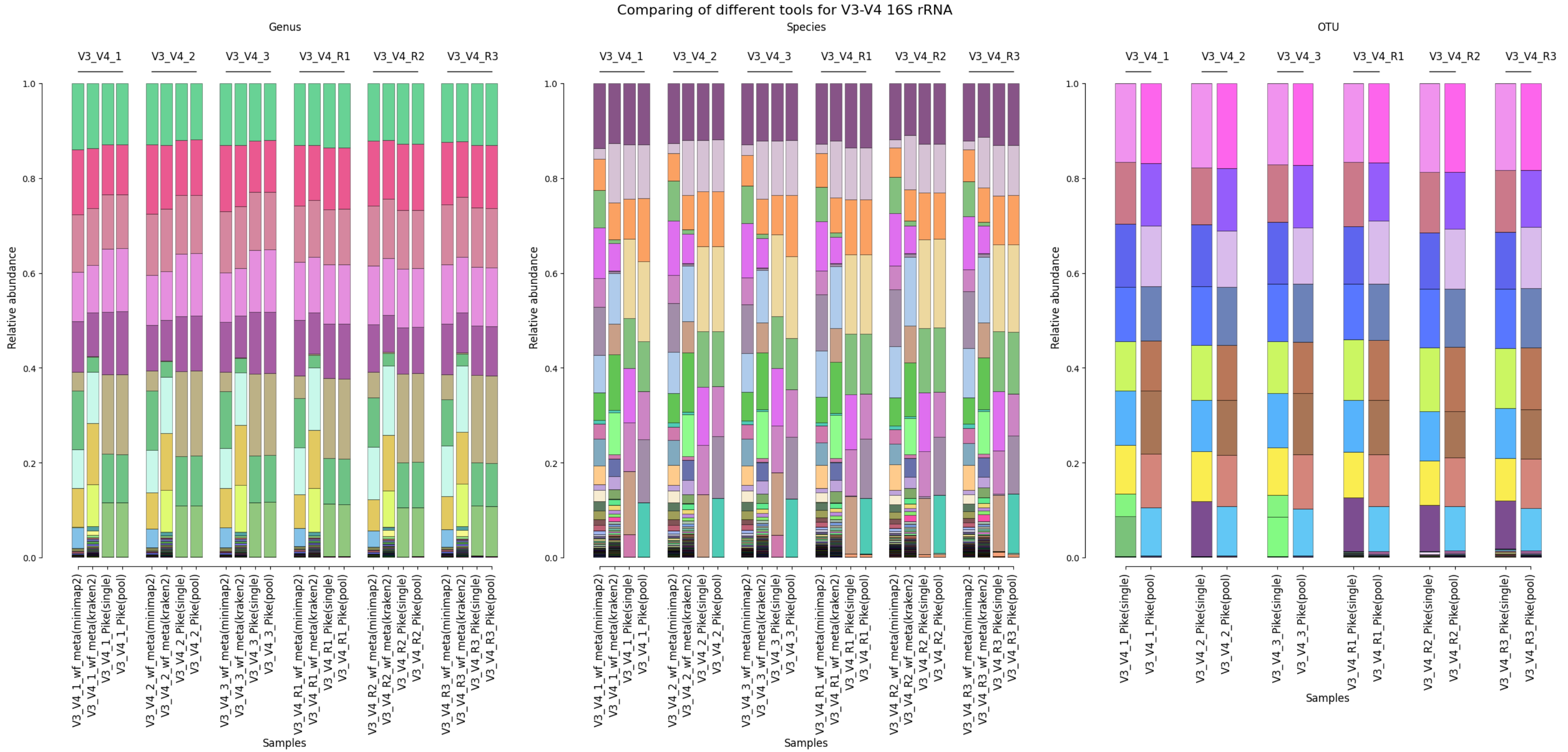

Supplementary 2 Fig.11 Results comparing different tools on our mock community array (V3-V4 16S rRNA). Each color corresponds to a unique taxon.

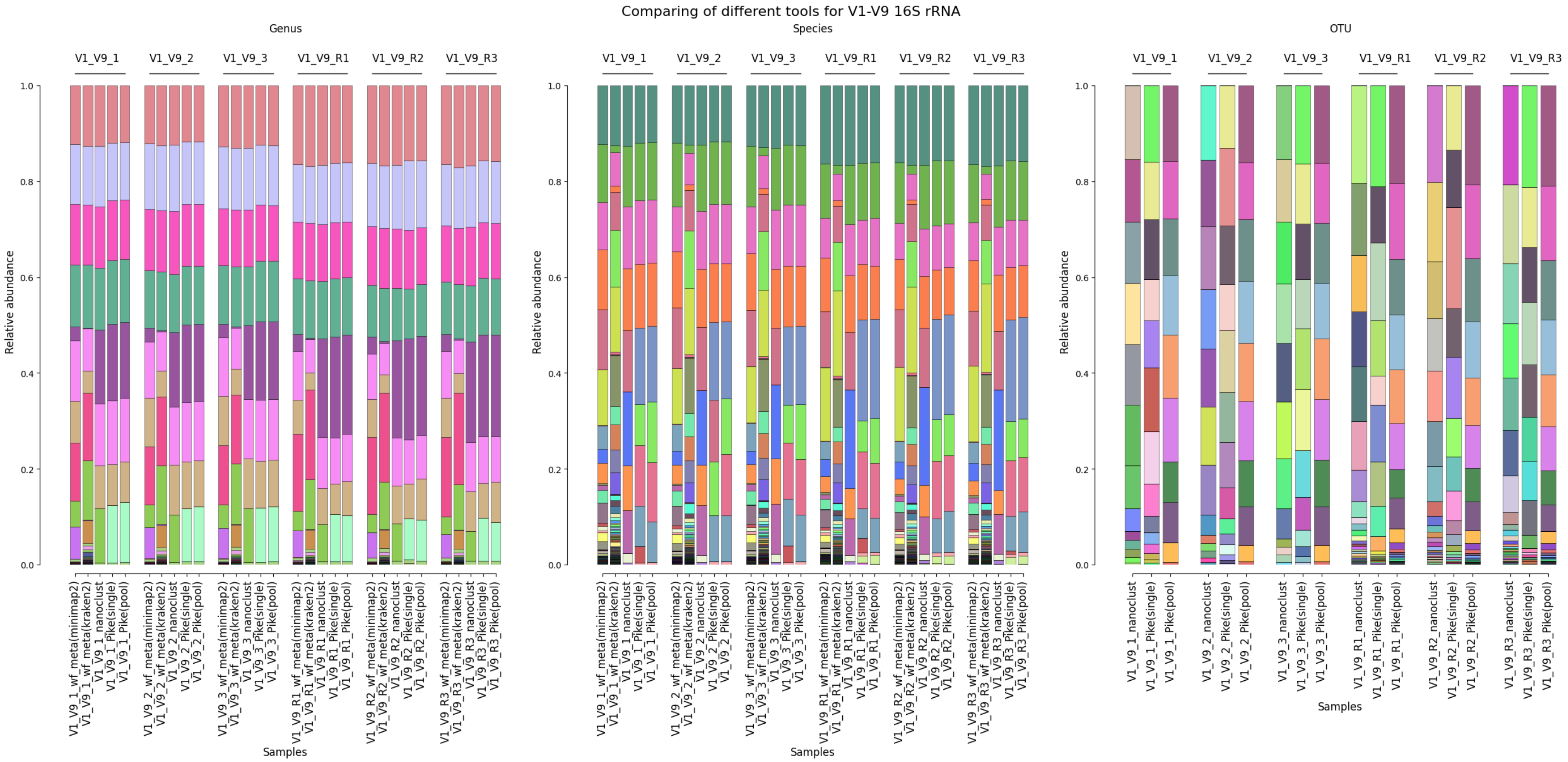

Supplementary 2 Fig.12 Results comparing different tools on our mock community array (V1-V9 16S rRNA). Each color corresponds to a unique taxon.

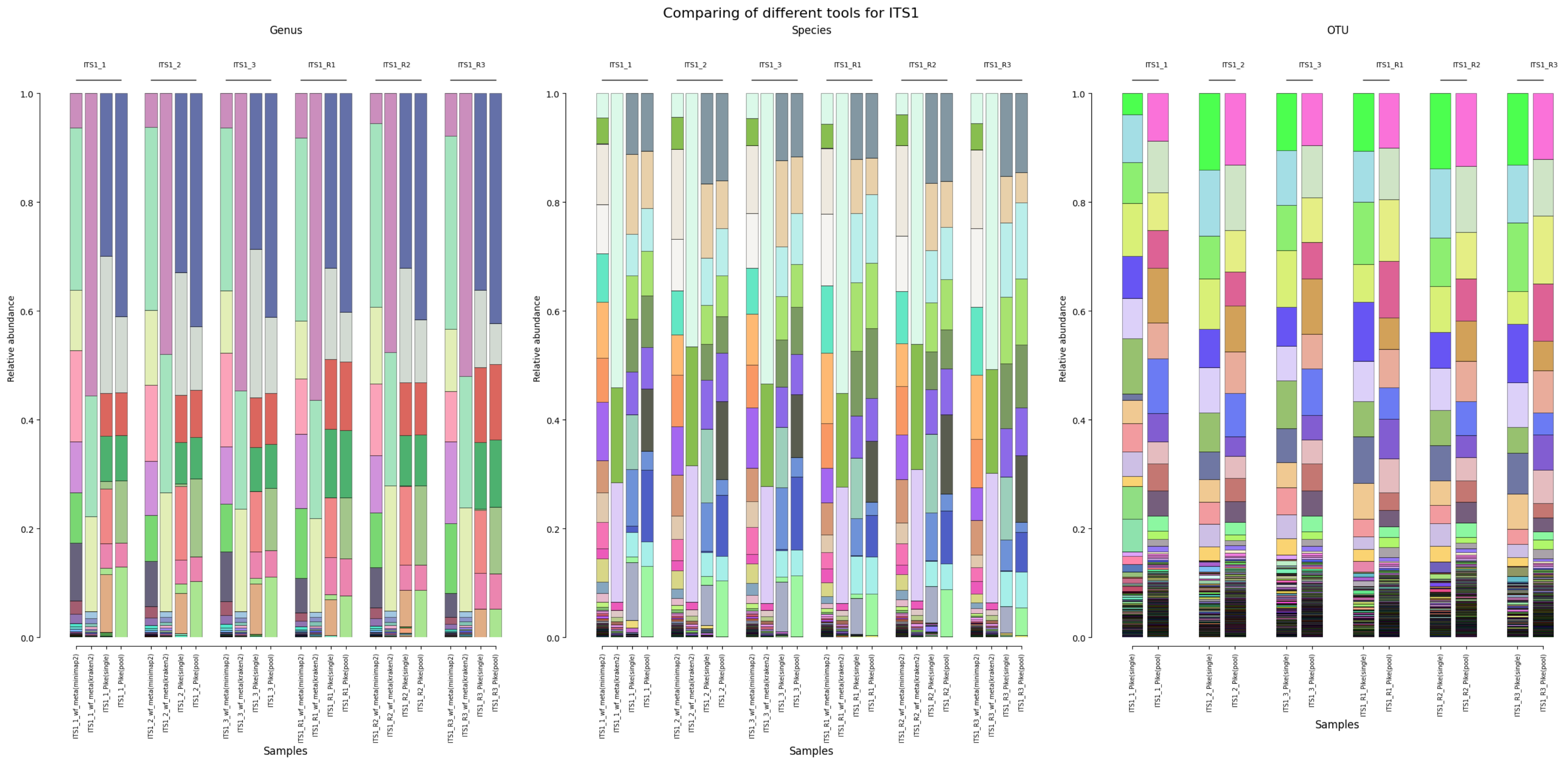

Supplementary 2 Fig.13 Results comparing different tools on our mock community array (ITS1). Each color corresponds to a unique taxon.

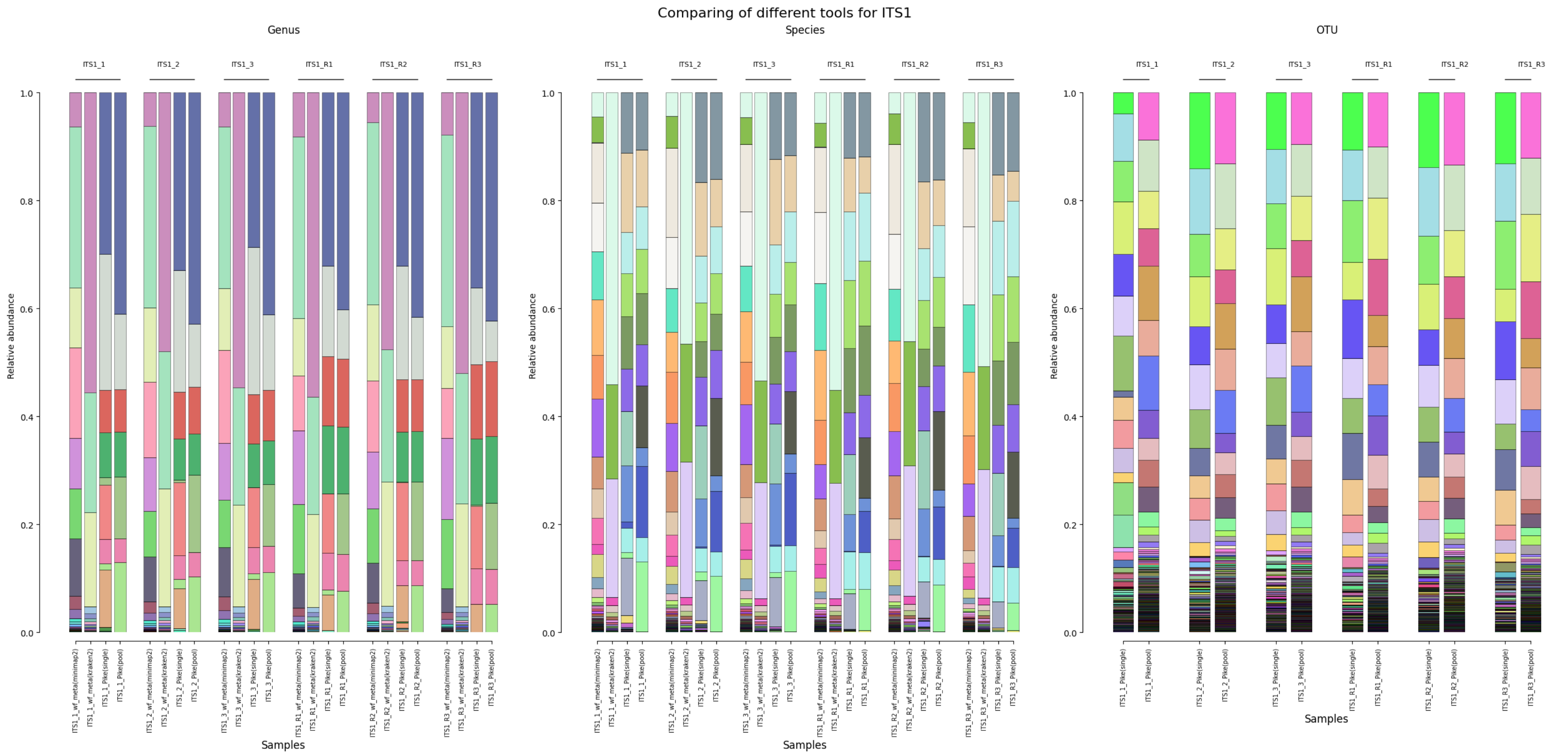

Supplementary 2 Fig.14 Results comparing different tools on our mock community array (ITS2). Each color corresponds to a unique taxon.

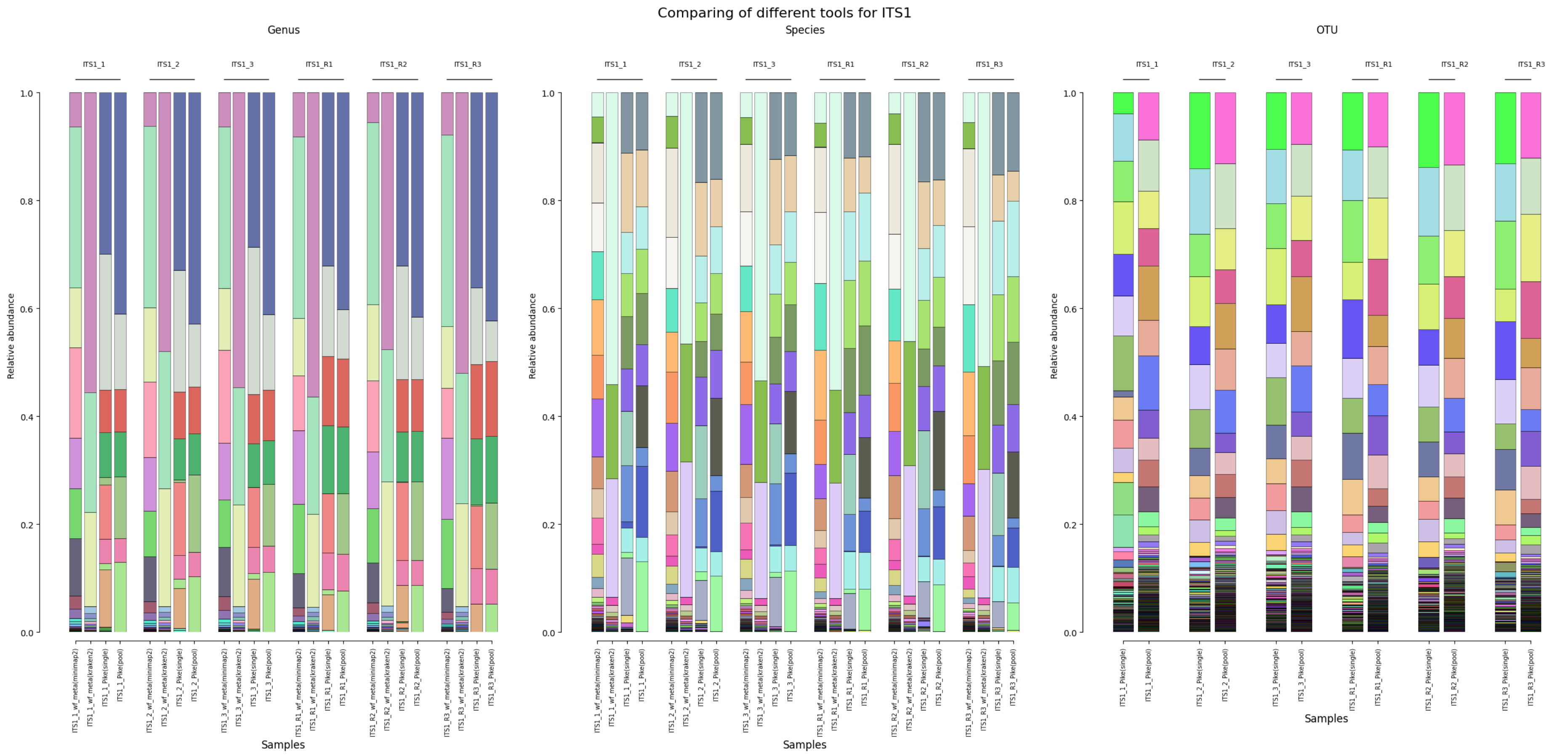

Supplementary 2 Fig.15 Results comparing different tools on our mock community array (ITS1-5.8S-ITS2). Each color corresponds to a unique taxon.

7. External nasopharyngeal swabs

To show that Pike was able to analyze real data, external data of nasopharyngeal swabs were downloaded [2]. The dataset contains a total of 59 complete 16SrRNA amplicon sequencing samples. However, in the work the authors considered only samples with a sequencing depth of more than 5000 reads per sample. We decided to perform a similar filtering of the samples, as a result of which 57 samples remained. It is also worth noting that this dataset was obtained using an old version of chemistry and a very old version of Guppy basecaller v3.2.10. For this reason, Pike was launched with reduced data quality requirements (pike -mode single -fastq porechop/ --trim_primer -primerF AGAGTTTGATCMTGGCTCAG -primerR CGGTTACCTTGTTACGACTT -threads 64 -output pike-res4 -minlen 900 -maxlen 1600 -output TEST_TRIM_2 -read_q_score 7 -letter_Q_lim 7). It is also worth noting that the reads were pre-filtered using porechop v0.2.4. The results were visualized in Supplementary 2 Fig.16.

In the case of real bacterial communities it is quite difficult to assess the correctness of the results obtained. However, it is worth noting that the main majors in our results were consistent with the results presented in the reference publication [2]. This analysis also shows that Pike is capable of analyzing older nanopore sequencing data.

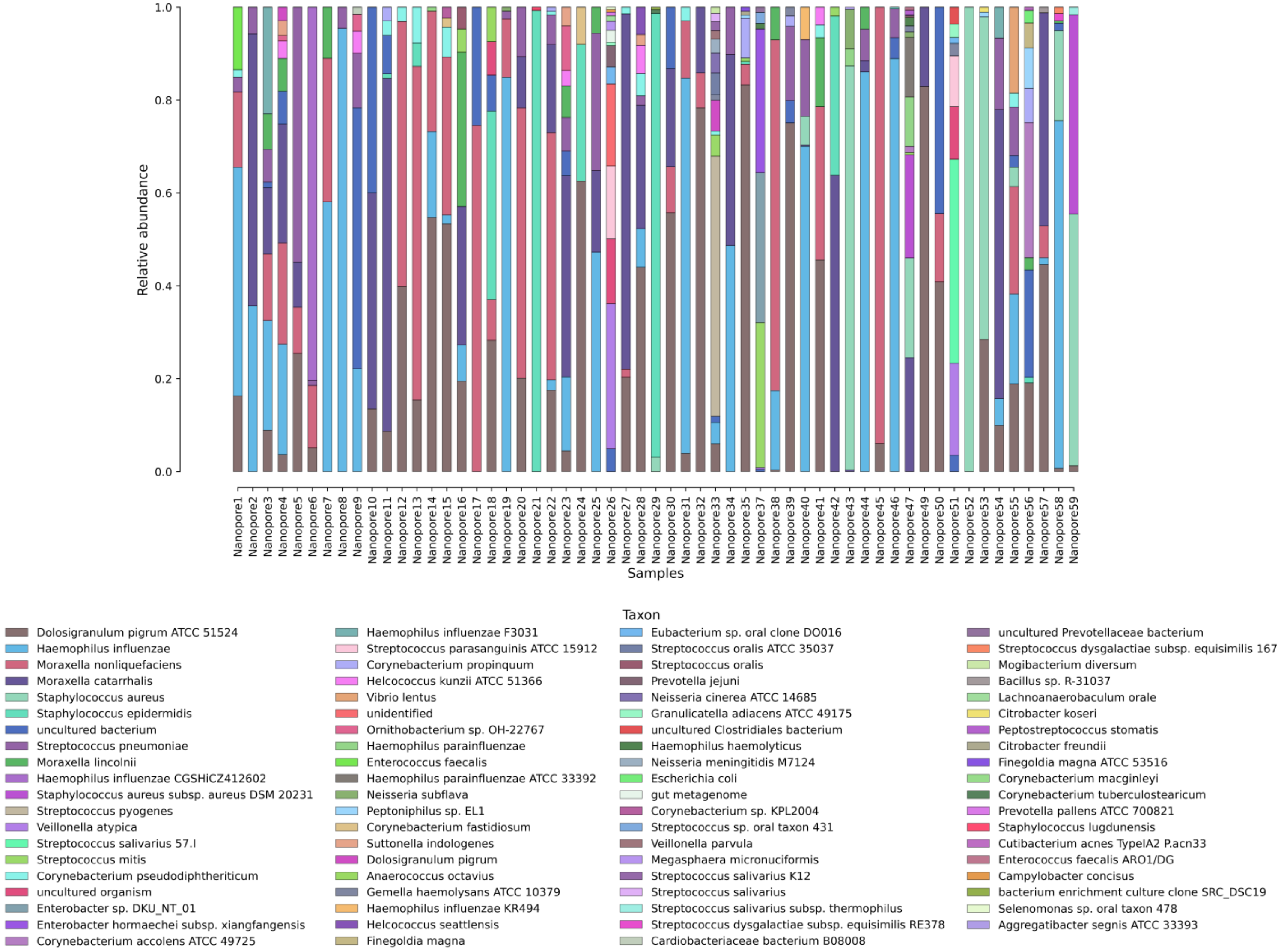

Supplementary 2 Fig.16 Analysis of external complete 16S sequencing data from nasopharyngeal swabs.

References

1. Krehenwinkel, H., Wolf, M., Lim, J. Y., Rominger, A. J., Simison, W. B., & Gillespie, R. G. (2017). Estimating and mitigating amplification bias in qualitative and quantitative arthropod metabarcoding. Scientific reports, 7(1), 17668.

2. Heikema, A. P., Horst-Kreft, D., Boers, S. A., Jansen, R., Hiltemann, S. D., de Koning, W., ... & Hays, J. P. (2020). Comparison of illumina versus nanopore 16S rRNA gene sequencing of the human nasal microbiota. Genes, 11(9), 1105.
