# Supplementary material for: Pike: OTU-Level Analysis for Oxford Nanopore Amplicon Metagenomics
Source: Int J Mol Sci. 2025 Apr 28;26(9):4168. doi: 10.3390/ijms26094168 (PMC12071631; doi:10.3390/ijms26094168)
Supplement: Supplementary file 1 [file ijms-26-04168-s001.zip › Supplementary_NEW/Supplementary_1.pdf]

Supplementary 1. Interesting cases

1. **The case of incorrect taxonomy definition in the UNITE reference database.**

It is recommended that only the most recent versions of the UNITE database be applied, as some anomalies have been observed in the older version, UNITE_public_10.05.2021. We decided to describe one of them in detail.

During a detailed analysis of the data obtained, it was noted that *Candida auris* is almost completely absent from the resulting data array. At the same time, a significant proportion of the fungal community is represented by s__Clavispora_sp.The OTUs belonging to the *s__Clavispora_sp* taxonomy were separately aligned to the nr/nt database using BLASTn. As a result, the OTUs corresponding to *s__Clavispora_sp* were determined to be indeed Candida auris (Sequence ID: CP060377.1, Identities=100%, Coverage=99%).

We then elected to examine one of the records to which analogous OTUs were aligned. A comparable example of an OTU and the corresponding record are provided in Supplementary Table 1 of UNITE. A separate alignment of record MW039128 to the nr/nt base using BLASTn also aligns to Candida auris (Sequence ID: CP147453.1,Identities=100%, Coverage=100%), indicating that the taxonomy recorded in UNITE is erroneous. The issue of erroneous taxonomy labeling in UNITE seems to be linked to collisions within the broader fungal taxonomy. This is verified by examining the NCBI taxonomy for Clavispora sp. (Fungi; Dikarya; Ascomycota; Saccharomyceta; Saccharomycotina; Saccharomycetes; Saccharomycetales; CUG-Ser1 clade; Metschnikowiaceae; Metschnikowiaceae). Incertae sedis; Candida/Metschnikowiaceae) and for Candida auris (Fungi; Dikarya; Ascomycota; Saccharomyceta; Saccharomycotina; Saccharomycetes; Saccharomycetales; CUG-Ser1 clade; Metschnikowiaceae).

Supplementary Table 1

OTU assembled by Pike	>Pike_OTU_Candida_auris<br>TAAGTTCAGCGGGTAGTCCTACCTGATTTGAGGCGACAACAAAACGAAAAAAAAGCGTAGATTTTTTCGTGCA<br>AGCTGTAATTTTGTGAATGCAACGCCACCGCGAAGATTGGTGAGAAGACATCACGCTCAAACAGGCATGCCTT<br>GGGGAATACCCCAAGGCGCAATGTGCGTTCAAAGATTCGATGATTCACGTCTGCAAGTCATACTACGTATCGC<br>ATTTCGCTGCGTTCTTCATCGATGCGAGAACCAAGAGATCCGTTGTTGAAAGTTTTATGTTTTTGTTTTAGTTG<br>AACTTAACGTTGGGTTAGTTTTAAAATCCAAATCAGTGTGTATGCAAAATATCAATAATGATCCTTCCGCAGG<br>TTCACCTACGGAAACCTTGTTACGACTTTTACTTCCATCCTCCGCTTATTGATATGCTTAAGTTCAGCGGGTAG<br>TCCTACCTGATTTGAGGCGACAACAAAACGAAAAAAAAGCGTAGATTTTTTCGTGCAAGCTGTAATTTTGTG<br>AATGCAACGCCACCGCGAAGATTGGTGAGAAGACATCACGCTCAAACAGGCATGCCTTGGGGAATACCCCAA<br>GGCGCAATGTGCGTTCAAAGATTCGATGATTCACGTCTGCAAGTCATACTACGTATCGCATTTCGCTGCGTTCT<br>TCATCGATGCGAGAACCAAGAGATCCGTTGTTGAAAGTTTTATGTTTTTGTTTTAGTTGAACTTAACGTTGGGT<br>TAGTTTTAAAATCCAAATCAGTGTGTATGCAAAATATCAATAATGATCCTTCCGCAGGTTCACCTACGGAAA
UNITE	>MW039128<br>k__Fungi;p__Ascomycota;c__Saccharomycetes;o__Saccharomycetales;f__Metschnikowiaceae;g__Clavispora;s__Clavispor<br>a_sp<br>TGTACACACCGCCCGTCGCTACTACCGATTGAATGGCTTAGTGAGGCCTCCGGATCTGGCATGCCCCGAGGGC<br>AACCTCGCCGCGCGCCGGAGAAGCTGGTCAAACTTGGTCATTTAGAGGAAGTAAAAGTCGTAACAAGGTTTC<br>CGTAGGTGAACCTGCGGAAGGATCATTATTGATATTTTGCATACACACTGATTTGGATTTTAAAACTAACCCA<br>ACGTTAAGTTCAACTAAAACAAAAACATAAAACTTTCAACAACGGATCTCTTGGTTCTCGCATCGATGAAGAA<br>CGCAGCGAAATGCGATACGTAGTATGACTTGCAGACGTGAATCATCGAATCTTTGAACGCACATTGCGCCTTG<br>GGGTATTCCCCAAGGCATGCCTGTTTGAGCGTGATGTCTTCTCACCAATCTTCGCGGTGGCGTTGCATTCACAA<br>AATTACAGCTTGCACGAAAAAAATCTACGCTTTTTTTTTCGTTTTGTTGTCGCCTCAAATCAGGTAGGACTACC<br>CGCTGAACTTAAGCATATCAATAAGCGGAGGAAAAGAAACCAACAGGGATTGCCTCAGTAACGGCGAGTGAA<br>GCGGCAAGAGCTCAACTTTGGAATCGCTCCGGCGAGTTGTAGTCTGGAGGTGGCCACCACGAGGTGTTCTAGC<br>AGCAGGCAAGTCCTTTGGAACAAGGCGCCAGCGAGGGTGACAGCCCCGTACCTGCTTTTGCTAGTGCTTCCTG<br>TGGCCCACCGACGAGTCGAGTTGTTTGGGAAT

Thus, we emphasize that the imperfection and ambiguity of fungal taxonomy can lead to ambiguous results and always requires additional verification using larger nr/nt basis sets.

2. Non-reference trim collisions.

In the course of analyzing the resulting array of reads, we observed a number of interesting characteristics of the reads obtained via the Oxford Nanopore platform. In particular, it was observed that a relatively large proportion of reads exhibited a complete or partial absence of the barcoding sequence. Furthermore, this phenomenon was predominantly observed on the read's right side. Despite the absence of a barcode sequence, the Guppi demultiplexer incorrectly identified a portion of the target sequence as a barcode sequence, resulting in the assignment of a relatively low barcode score. The distributions of the barcode score metrics were visualized in Supplementary Figure 1.

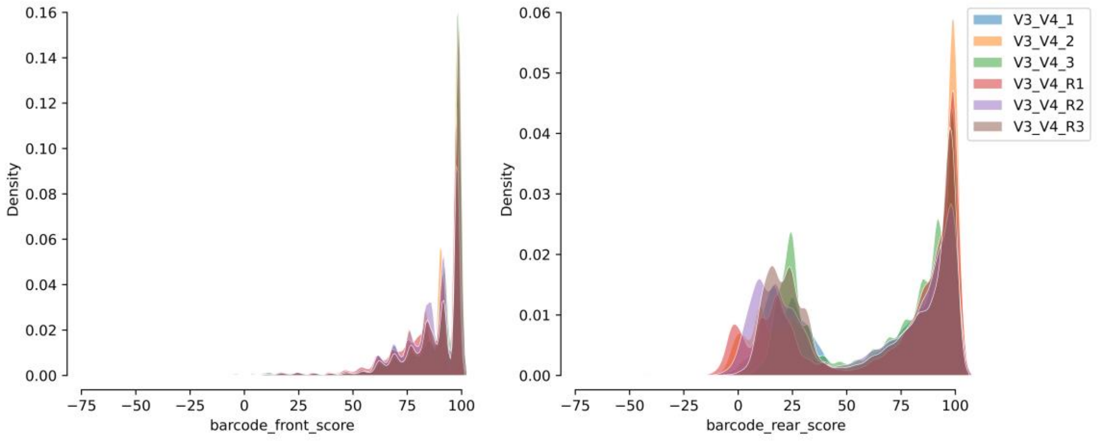

Supplementary 1 Fig.1. Distributions of barcode_score for the left barcode (left graph), right barcode (right graph).

Therefore, in the array under consideration for left reads, a unimodal distribution is observed, with a skew towards the maximum values. At the same time, reads for the right barcodes exhibit bimodal distributions. A more detailed analysis of the reads situated within the left peak revealed that these reads lack a right barcode. It was determined that for such reads, the conclusion regarding the barcode identity was solely based on the left side. When utilizing software to trim these reads, it is also possible to specify a mod with a forced search for barcodes at both ends.

Initially, it was assumed that in the resulting array, barcodes should be observed on both sides. It was further assumed that reads containing only one of the two barcoding sequences may be cut off too much on one of the sides. In our research, we initially employed the porechop program with the --require_two_barcodes option and standard barcode search parameters to trim barcodes. The analysis of the resulting array using Pike revealed a notable decline in the relative representation of *Lactococcus lactis* in the V3_V4_3 sample. Following an extensive investigation into the causes of the alteration in bacterial composition, it was determined that a considerable reduction in *Lactococcus lactis* reads occurred during the process of removing the reverse primer. Furthermore, the findings were validated through analysis with wf-metagenomic, which substantiated that the observed anomaly was a direct consequence of trimming the reverse primer.

We were unable to definitively determine why porechop mistakenly cuts off part of the *Lactococcus lactis* sequence. However, no similar problems were found after using guppi_barcode.

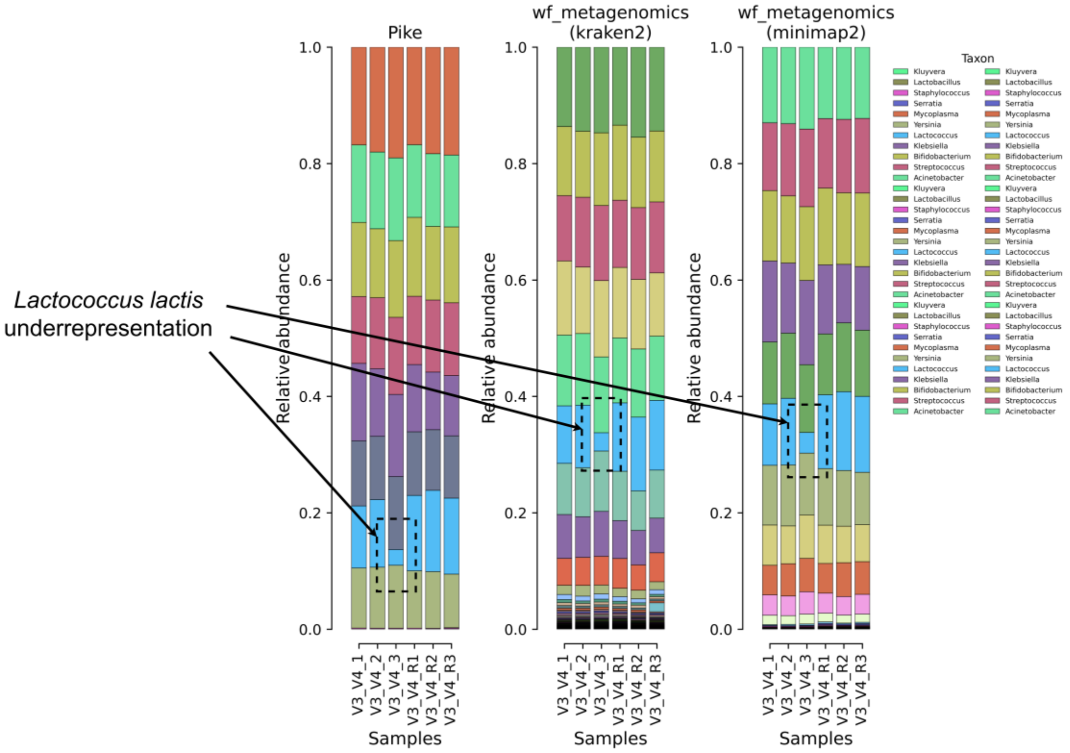

Supplementary 1 Fig.2. Results of data analysis after porechop trimming and cutting of primer sequences (primers were cut off forcefully on both sides).
