# Supplementary material for: Pike: OTU-Level Analysis for Oxford Nanopore Amplicon Metagenomics
Source: Int J Mol Sci. 2025 Apr 28;26(9):4168. doi: 10.3390/ijms26094168 (PMC12071631; doi:10.3390/ijms26094168)
Supplement: Supplementary file 1 [file ijms-26-04168-s001.zip › Supplementary_NEW/Supplementary_3.pdf]

Supplementary 3. Additional figures.

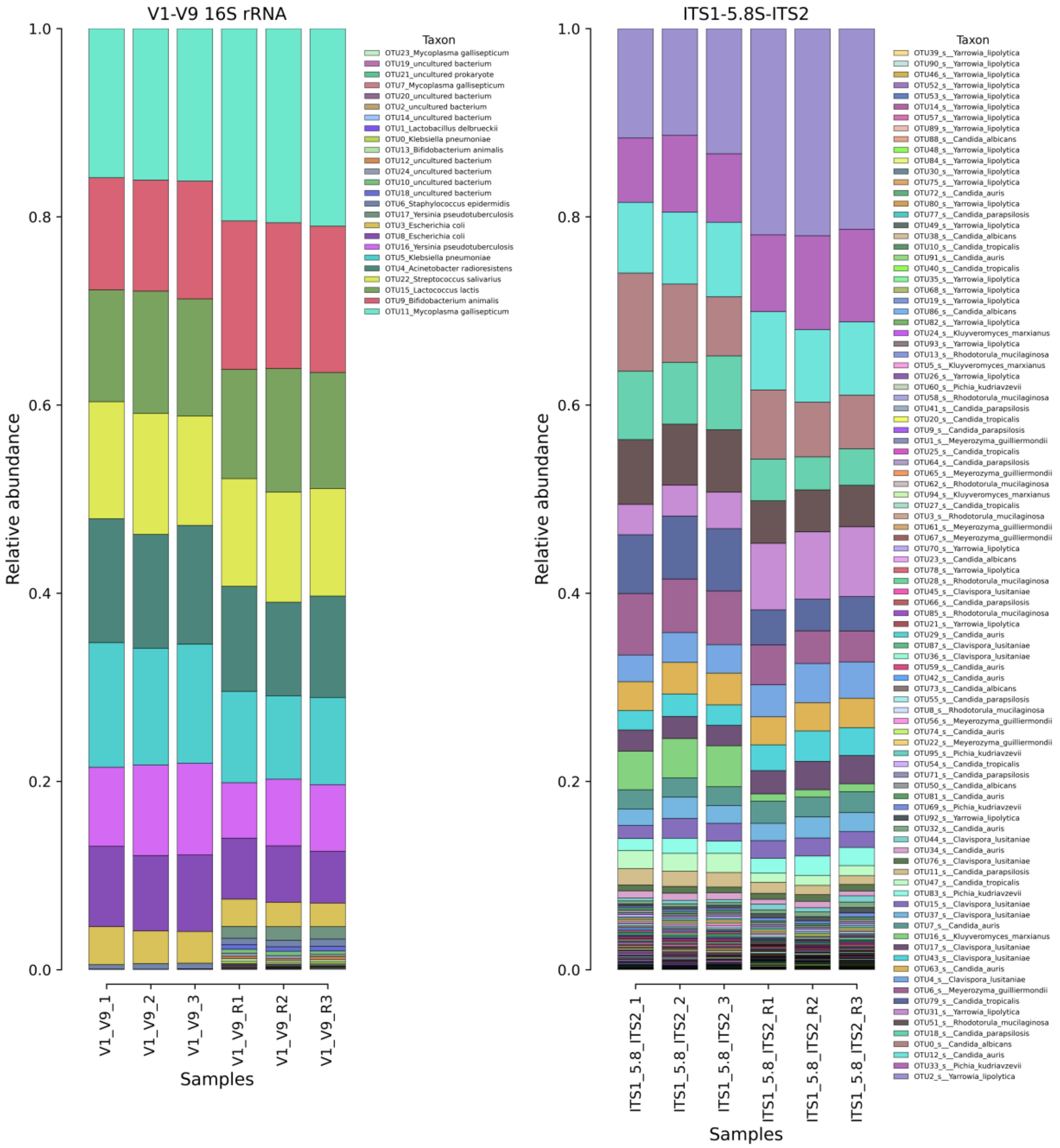

Supplementary 3 Fig.1 Barplot of Pike's results from long amplicon sequencing data (V1-V9 and ITS1-5.8S-ITS2).

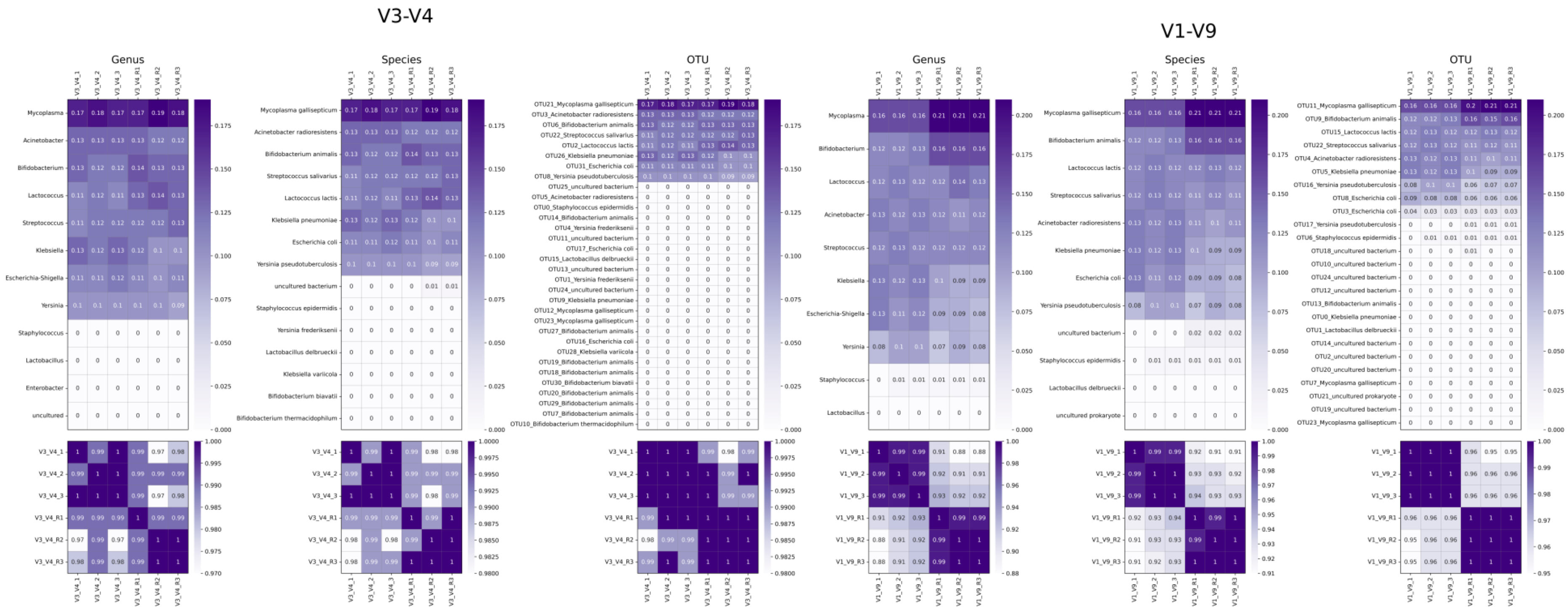

Supplementary 3 Fig.2 Pike bacterial mock community processing results with pool mode. Upper figures - results of assessing relative representation at different taxonomy levels. The lower pictures are the values of the Spearman rank correlation coefficient. Zero values in the picture correspond to values < 0.001.

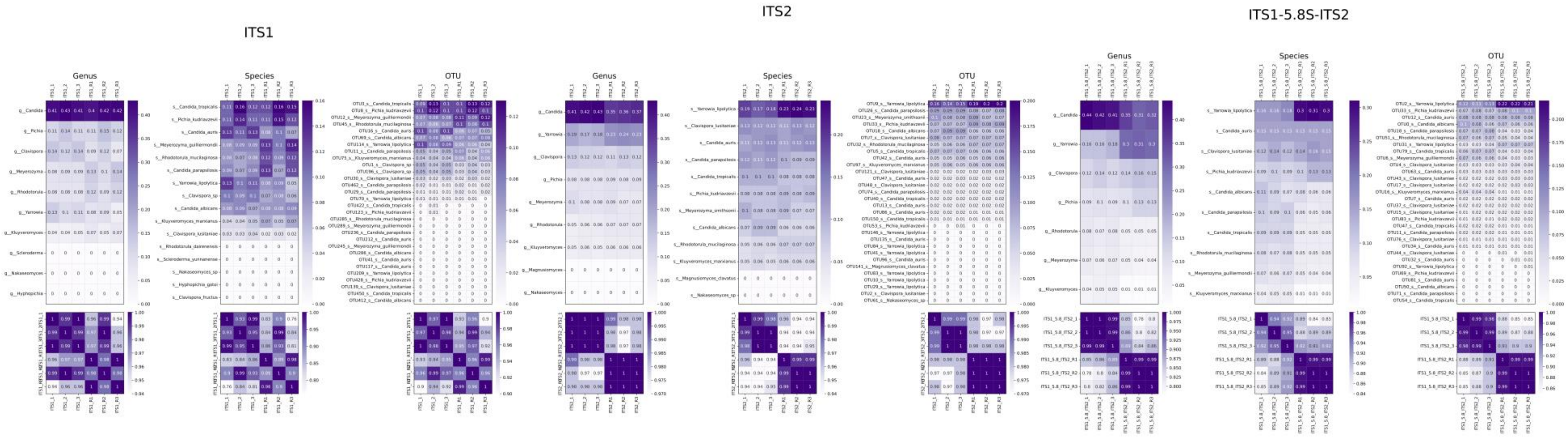

Supplementary 3 Fig.4 Pike fungal mock community processing results with pool mode. Upper figures - results of assessing relative representation at different taxonomy levels. The lower pictures are the values of the Spearman rank correlation coefficient. Zero values in the picture correspond to values < 0.001.

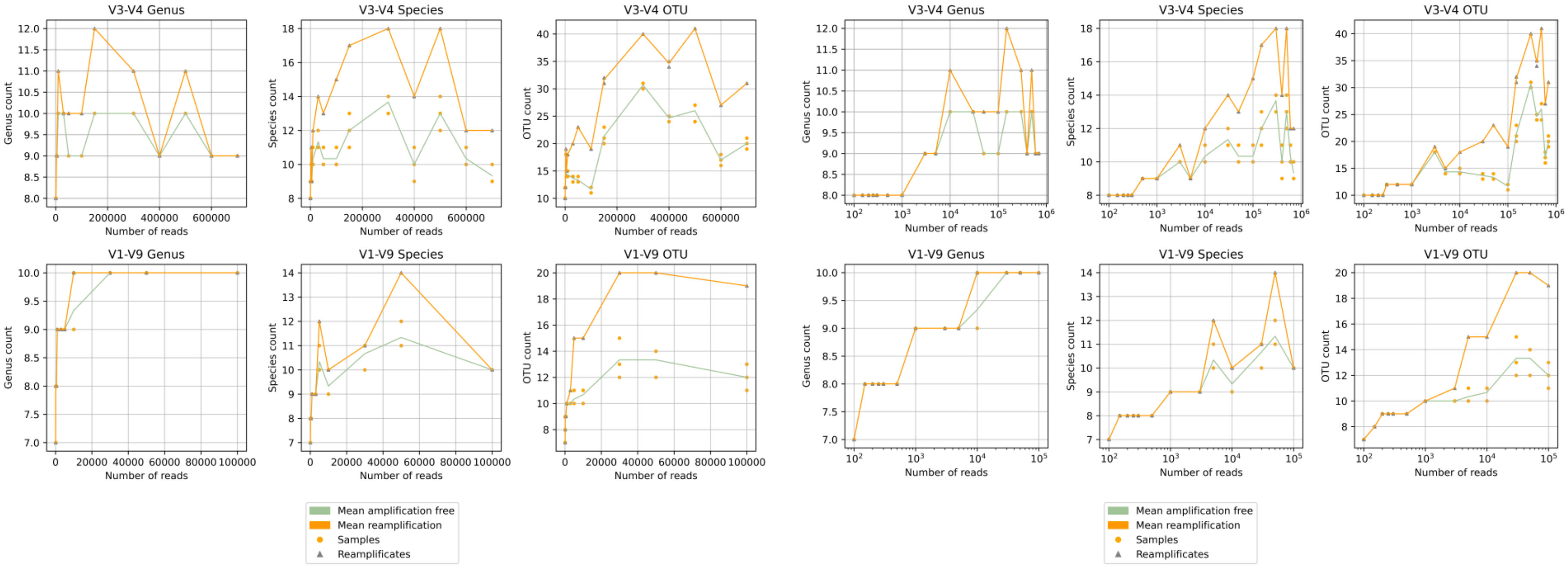

Supplementary 3 Fig.5 Rarefaction curves for 16S mock community data (left - original, right - log scale). Pike **pool mode** results.

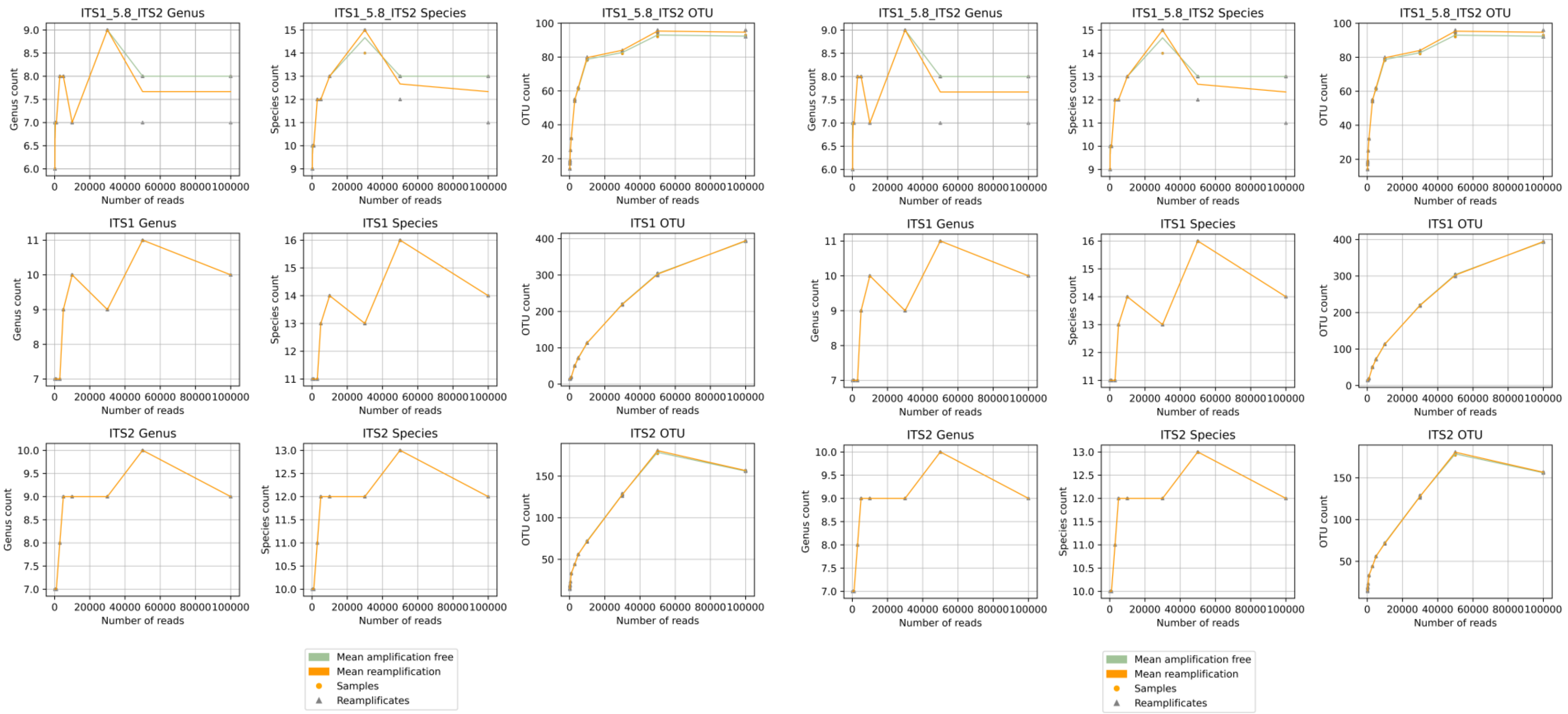

Supplementary 3 Fig.6 Rarefaction curves for ITSs mock community data (left - original, right - log scale). Pike **pool mode** results.

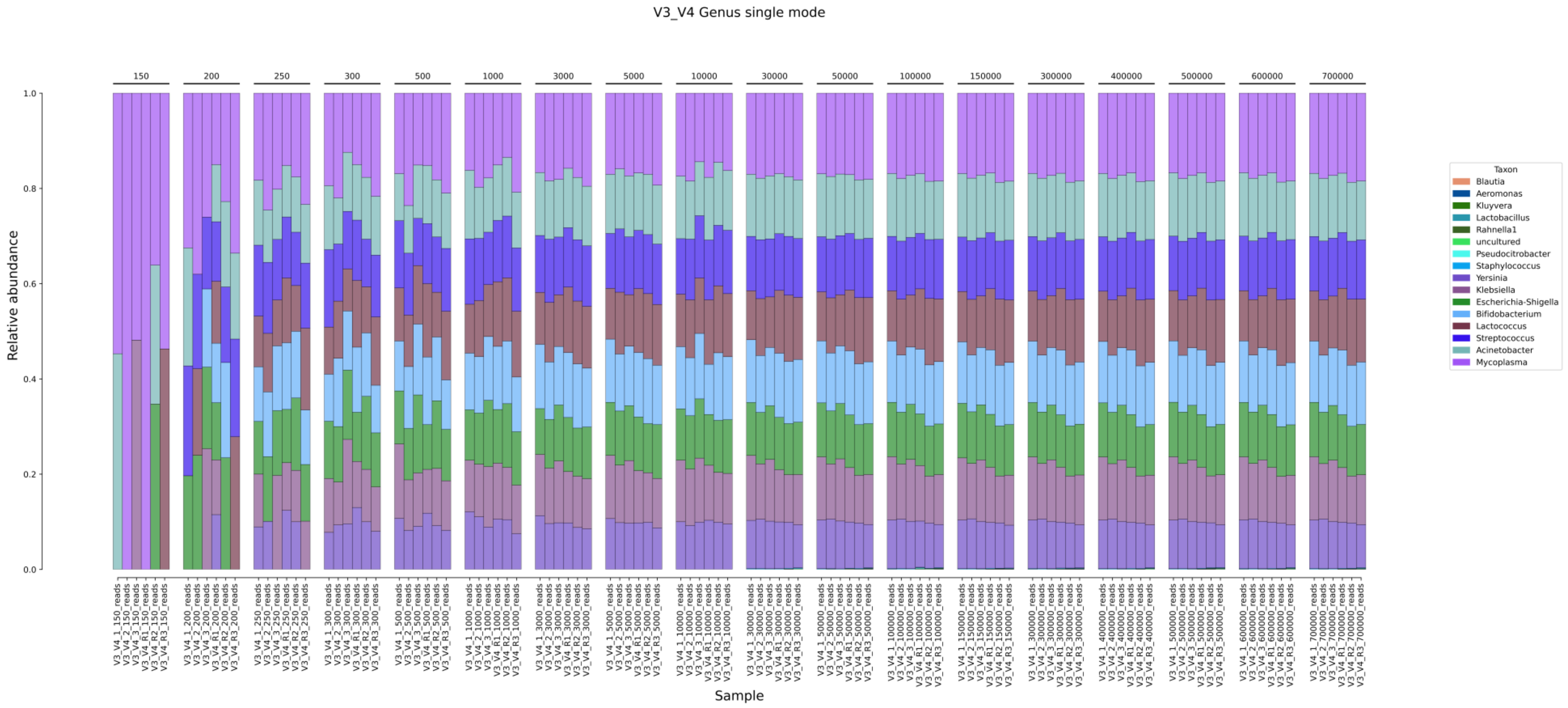

Supplementary 3 Fig.7 Rarefaction results for individual read arrays for V3-V4 (Genus level, **single mode**).

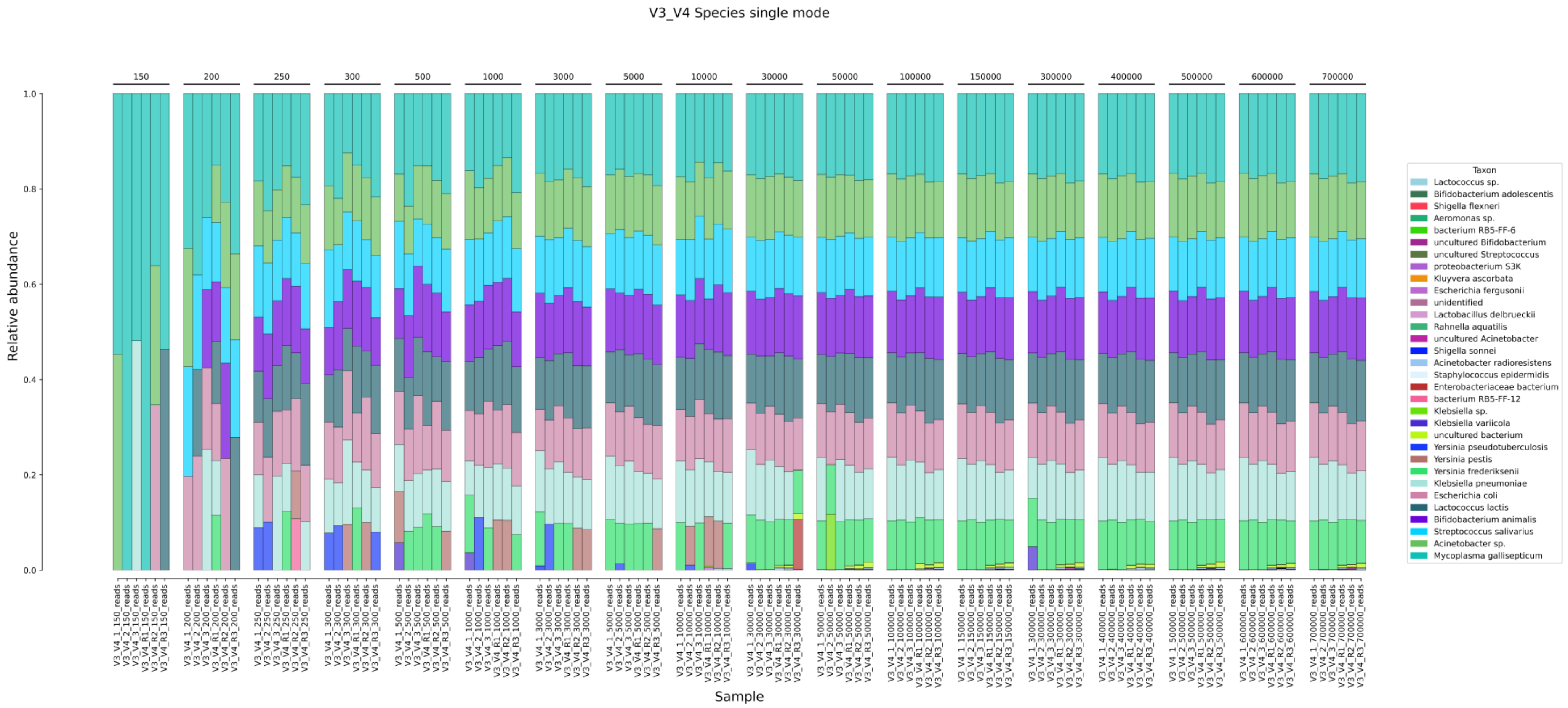

Supplementary 3 Fig.8 Rarefaction results for individual read arrays for V3-V4 (Species level, **single mode**).

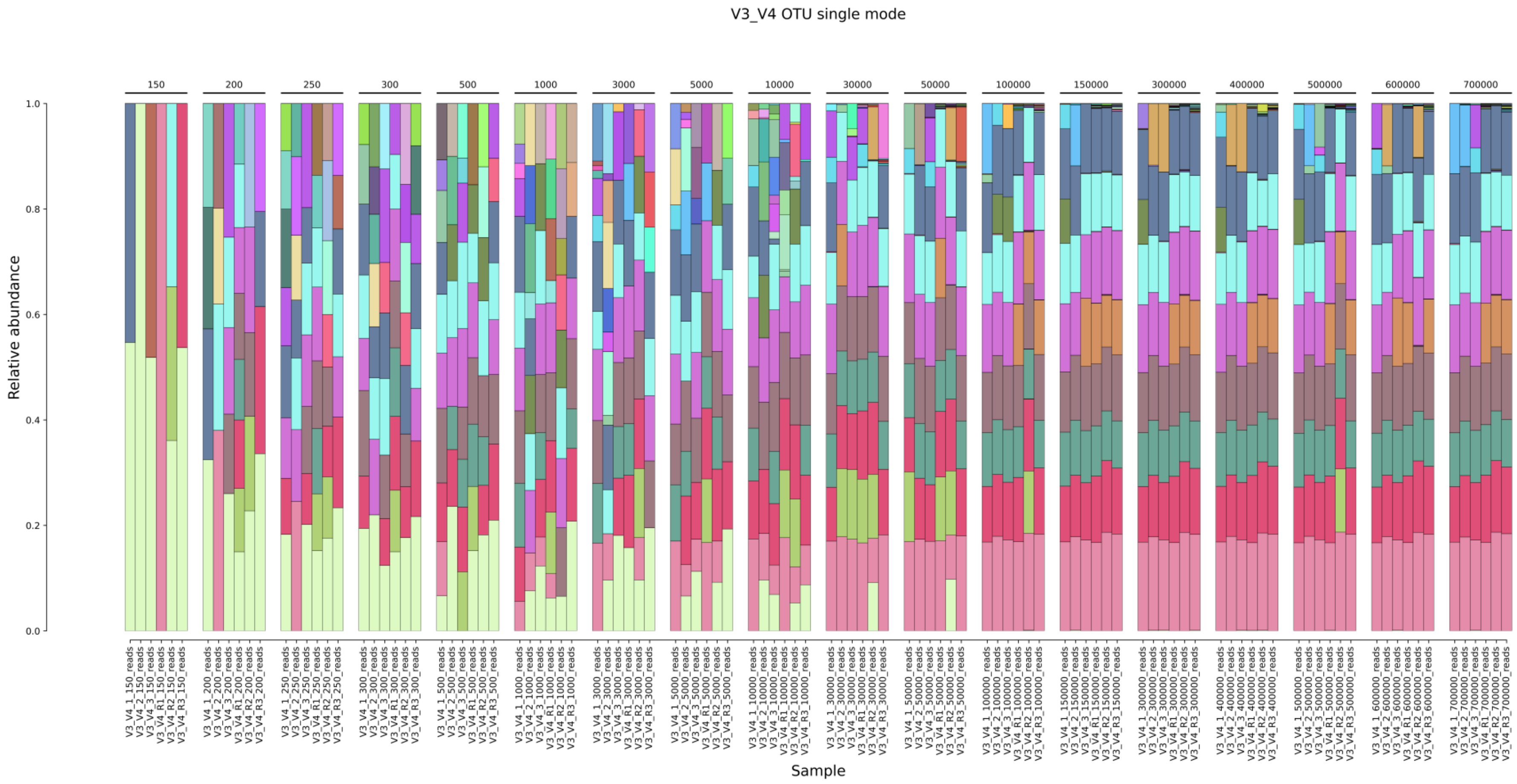

Supplementary 3 Fig.9 Rarefaction results for individual read arrays for V3-V4 (OTU level, **single mode**).

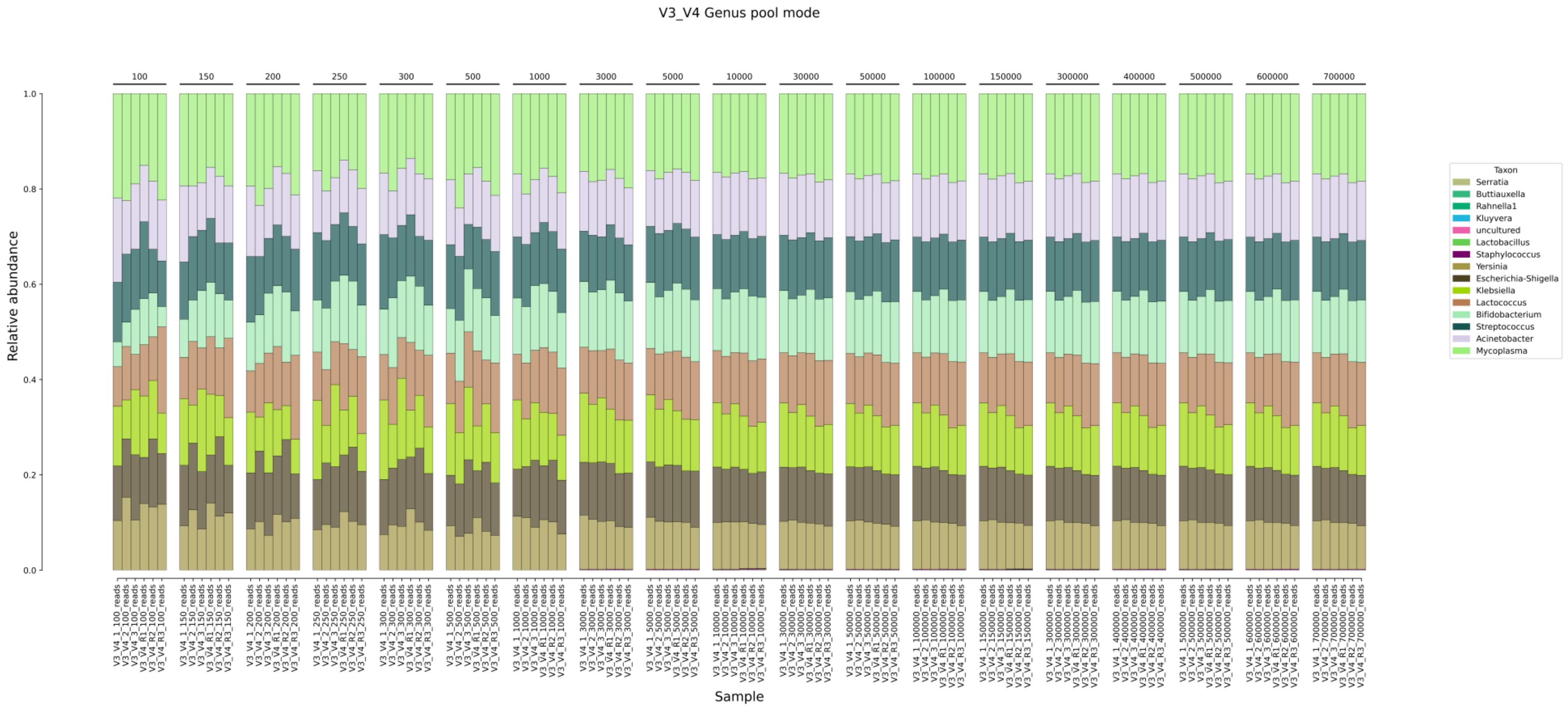

Supplementary 3 Fig.10 Rarefaction results for individual read arrays for V3-V4 (Genus level, **pool mode**).

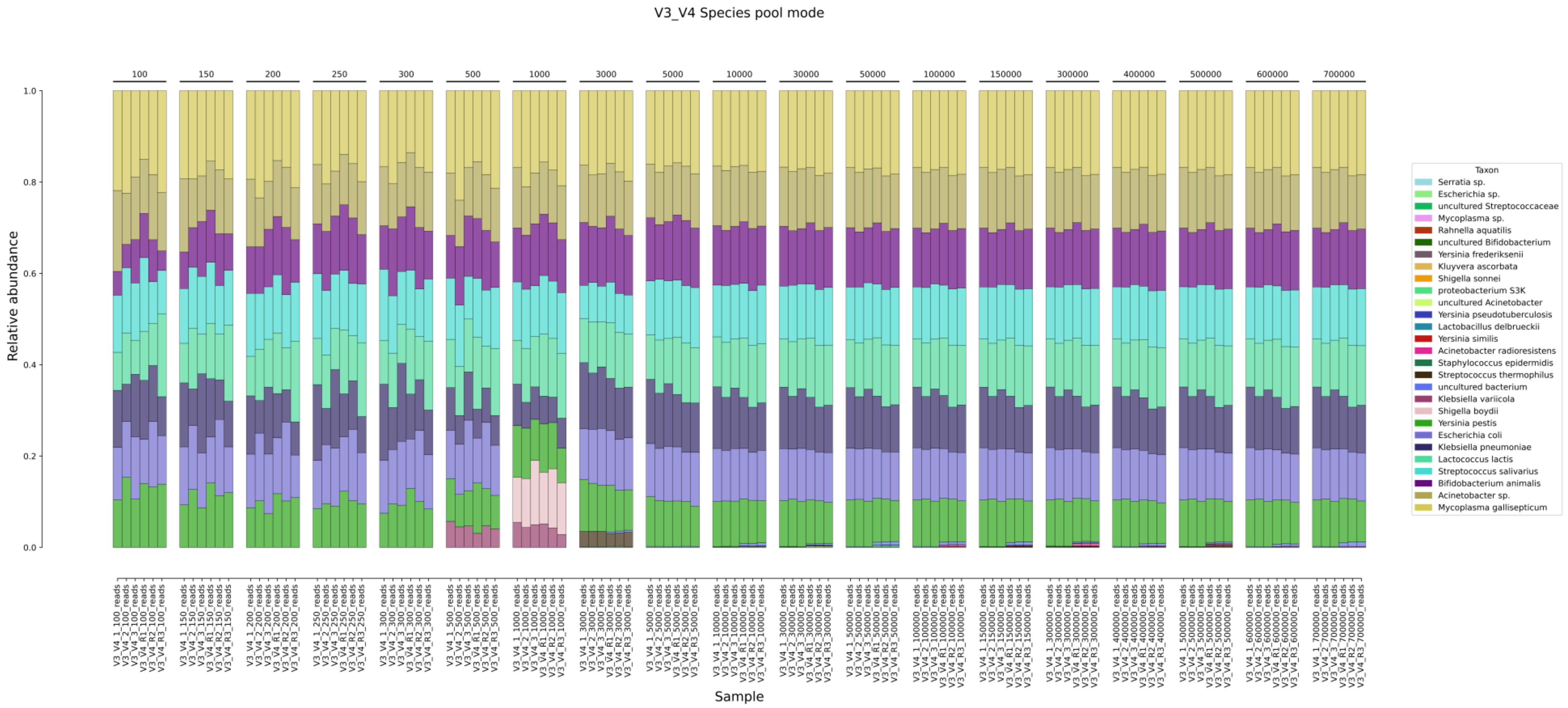

Supplementary 3 Fig.11 Rarefaction results for individual read arrays for V3-V4 (Species level, **pool mode**).

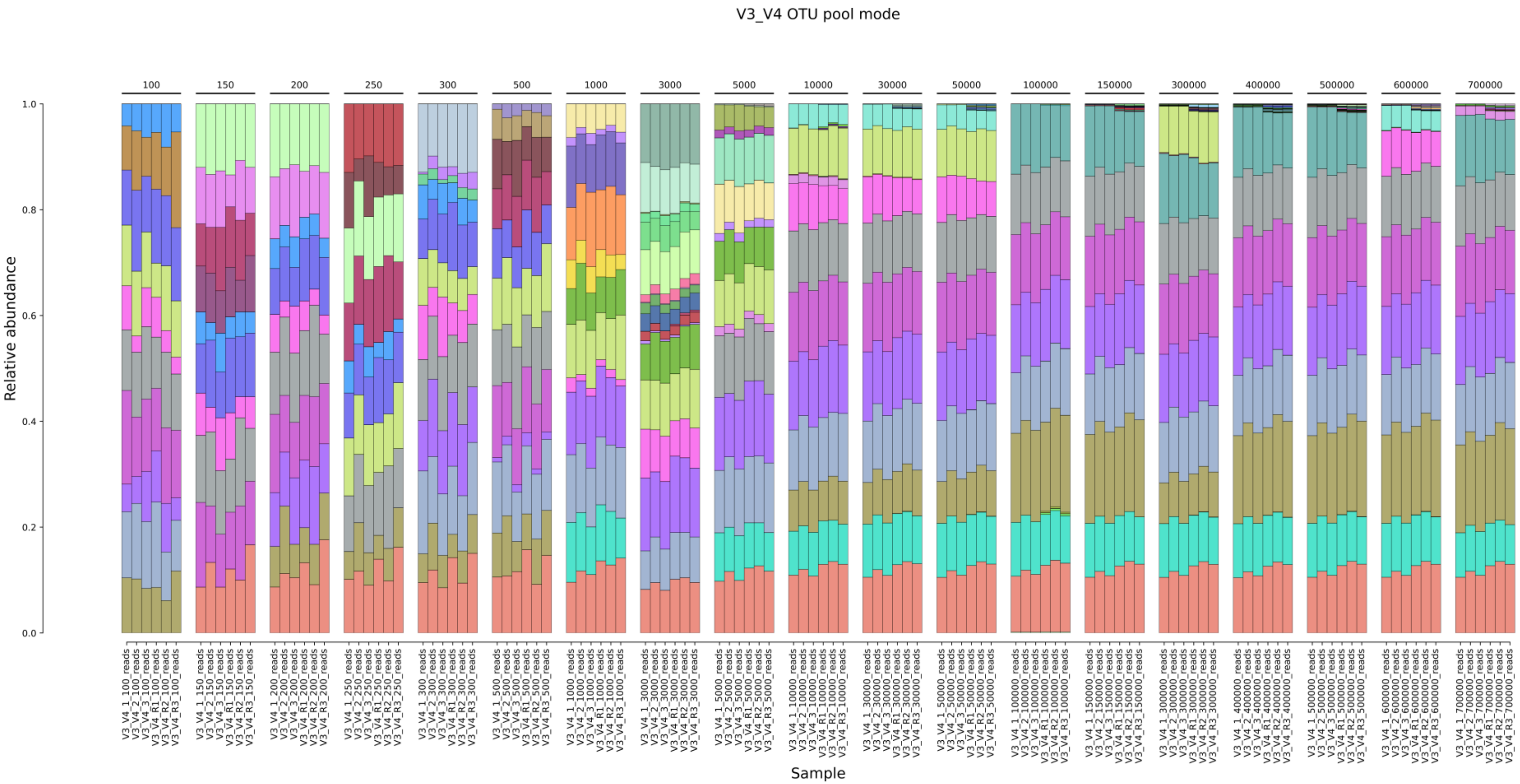

Supplementary 3 Fig.12 Rarefaction results for individual read arrays for V3-V4 (OTU level, **pool mode**).

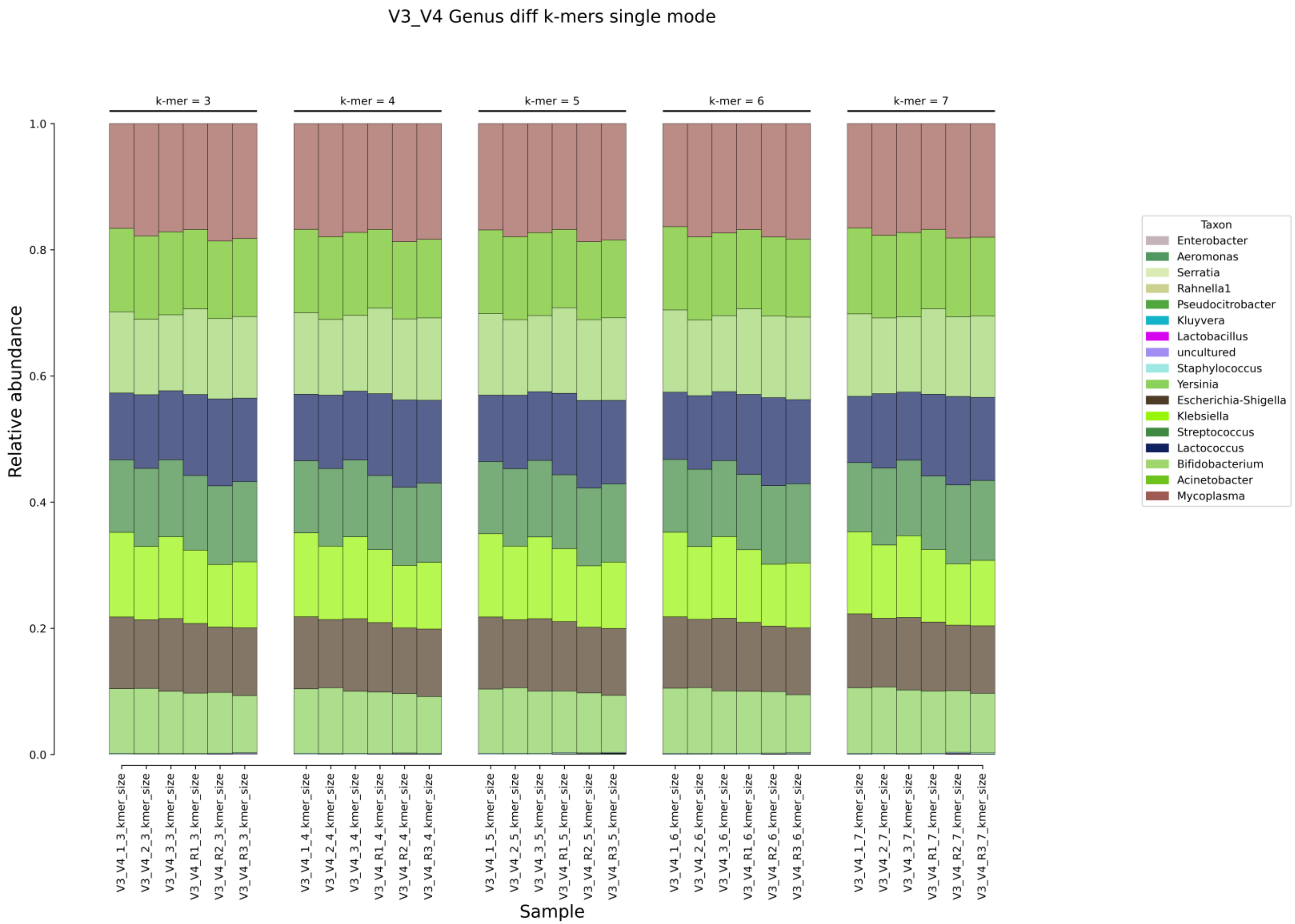

Supplementary 3 Fig.13 Pike's results for V3-V4 with different K-measure sizes (Genus level, **single mode**).

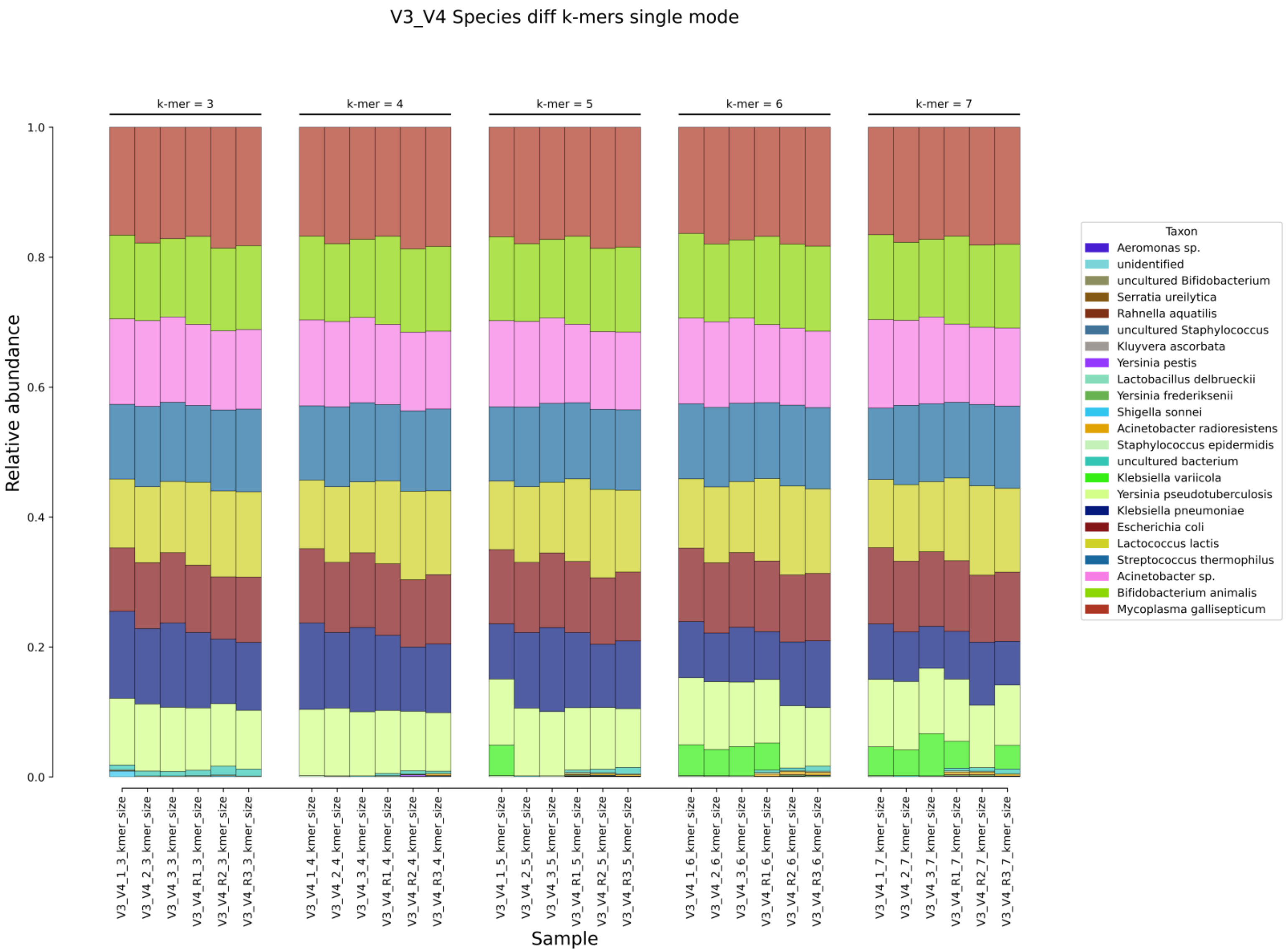

Supplementary 3 Fig.14 Pike's results for V3-V4 with different K-measure sizes (Species level, **single mode**).

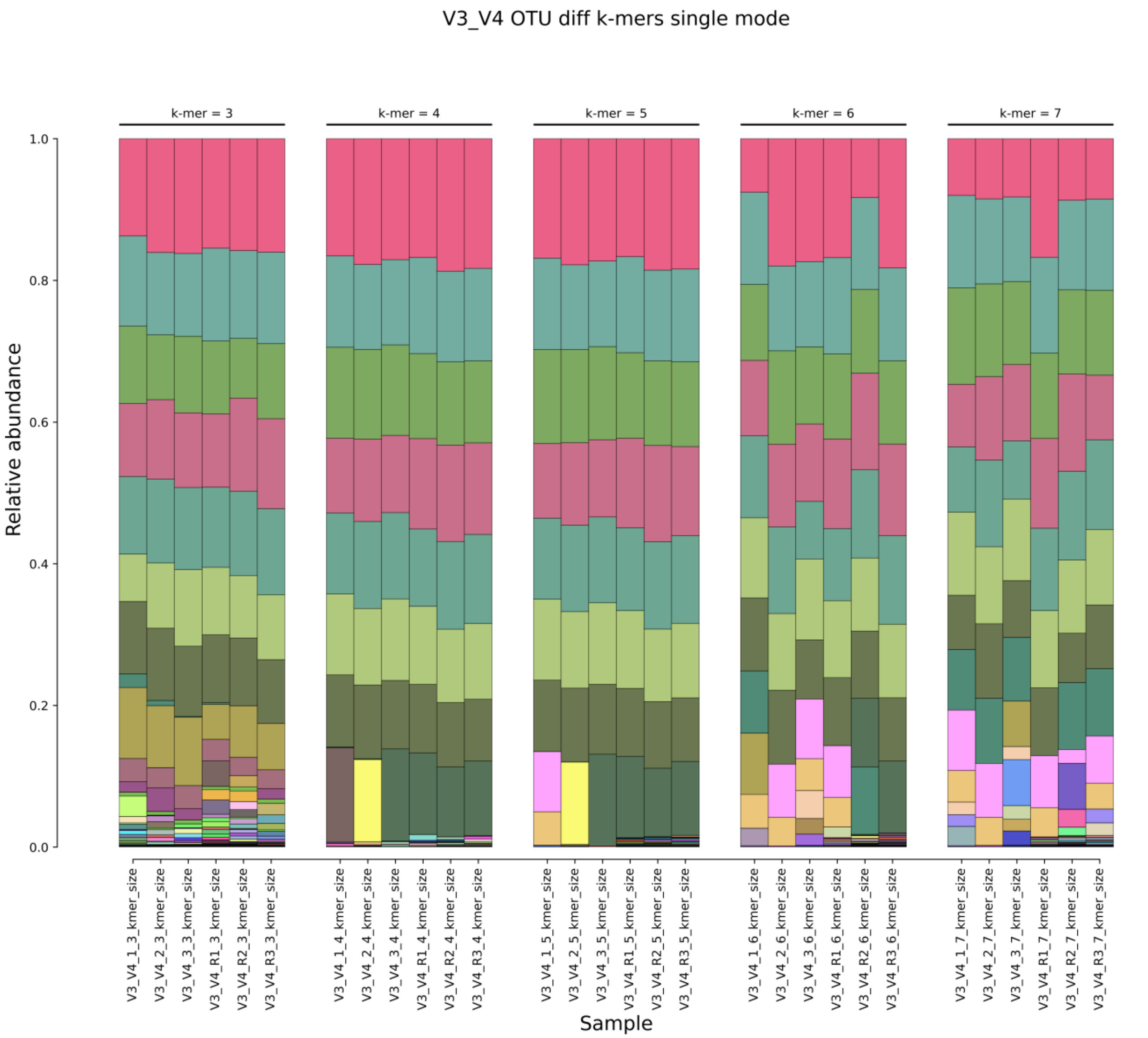

Supplementary 3 Fig.15 Pike's results for V3-V4 with different K-measure sizes (OTU level, **single mode**).

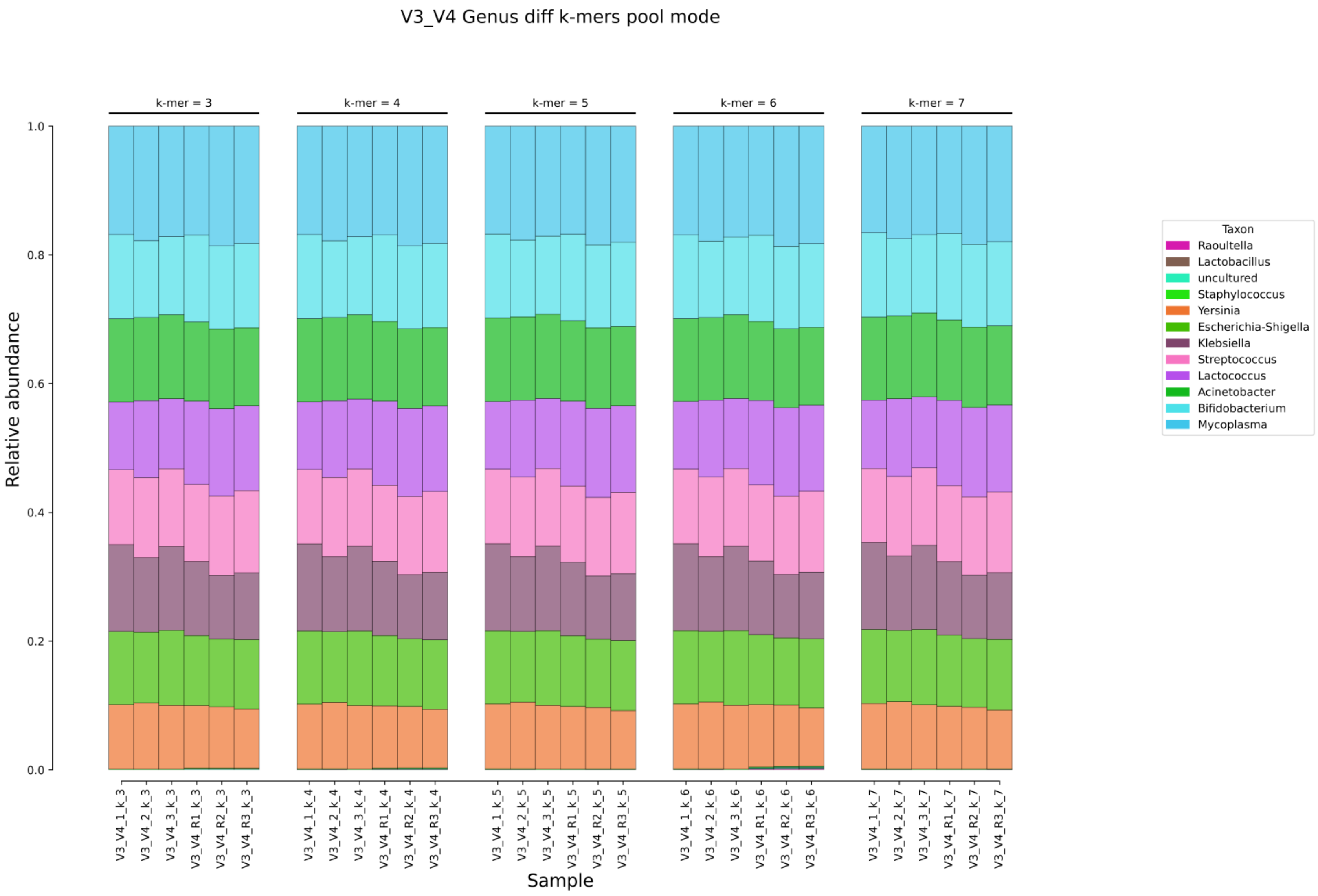

Supplementary 3 Fig.16 Pike's results for V3-V4 with different K-measure sizes (Genus level, **pool mode**).

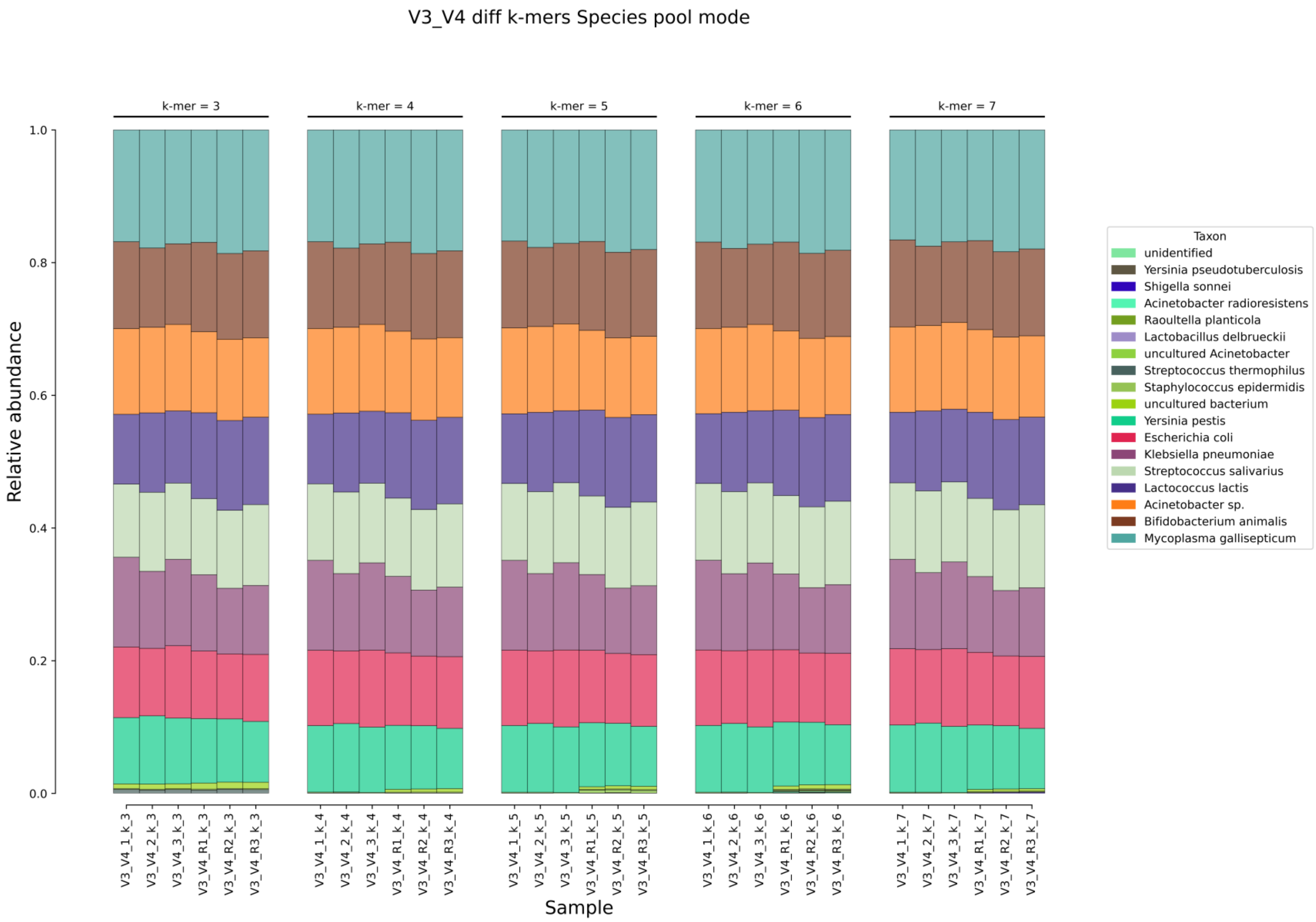

Supplementary 3 Fig.17 Pike's results for V3-V4 with different K-measure sizes (Species level, **pool mode**).

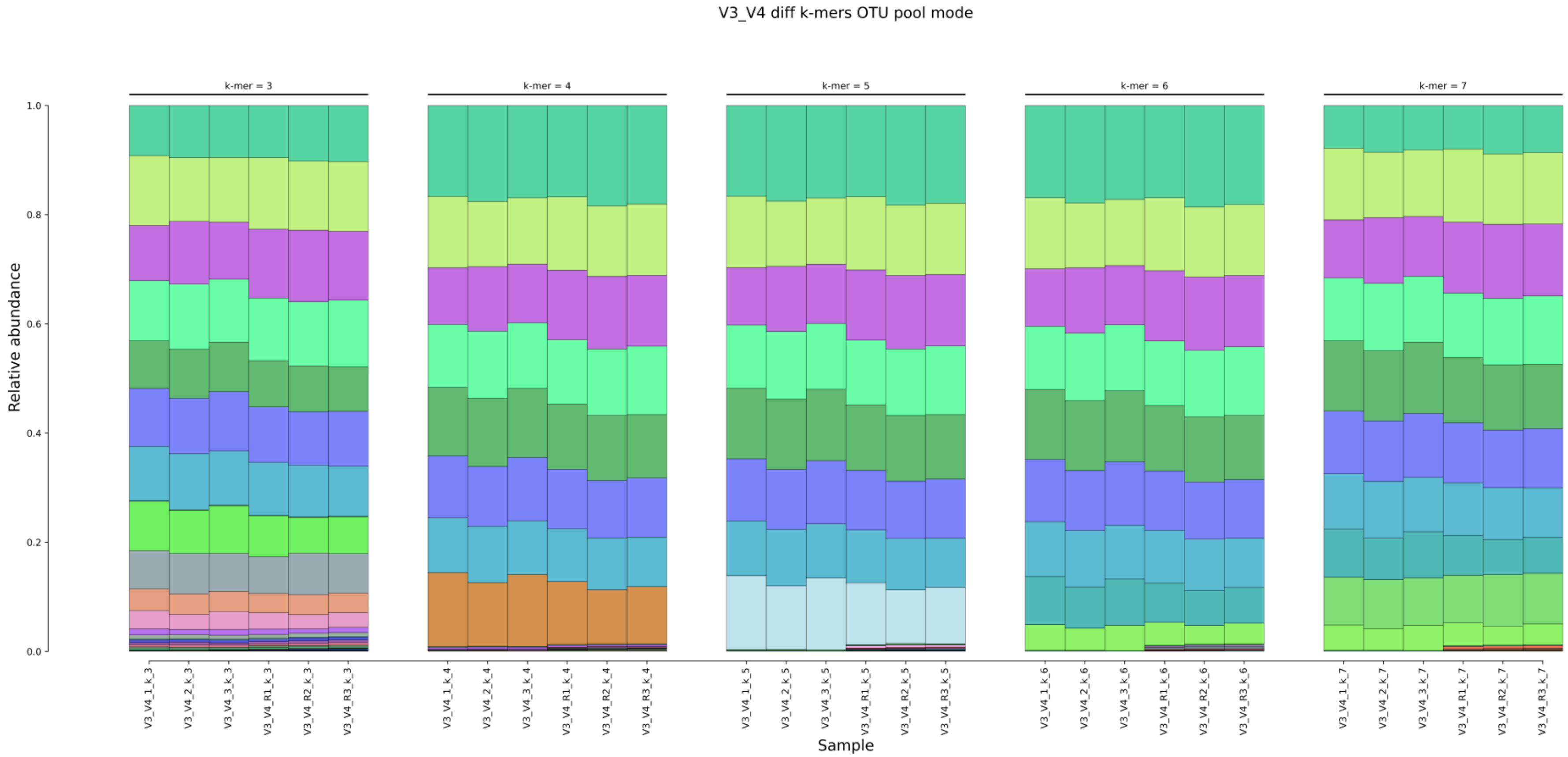

Supplementary 3 Fig.18 Pike's results for V3-V4 with different K-measure sizes (OTU level, **pool mode**).

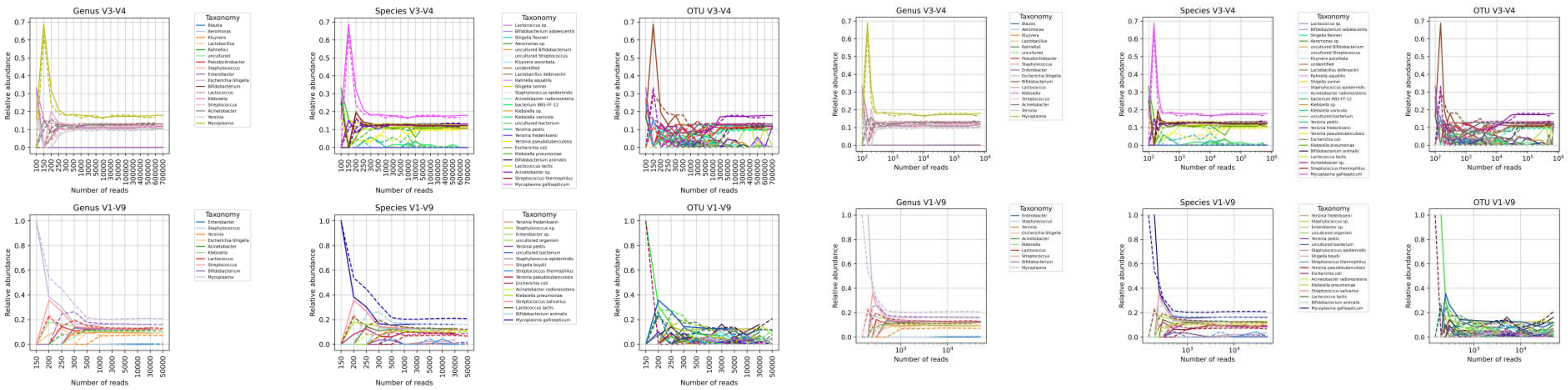

Supplementary 3 Fig.19 Change in relative representation as a function of the number of input reads (left - original, right - log scale). Bacterial community, **single mode.**

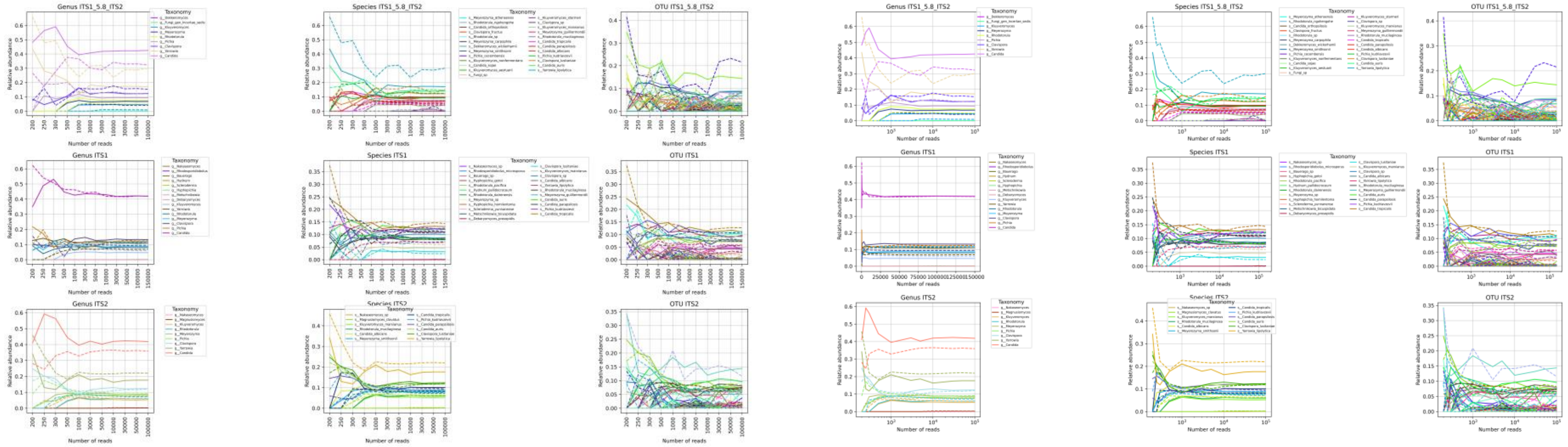

Supplementary 3 Fig.20 Change in relative representation as a function of the number of input reads (left - original, right - log scale). Fungal community, **single mode.**

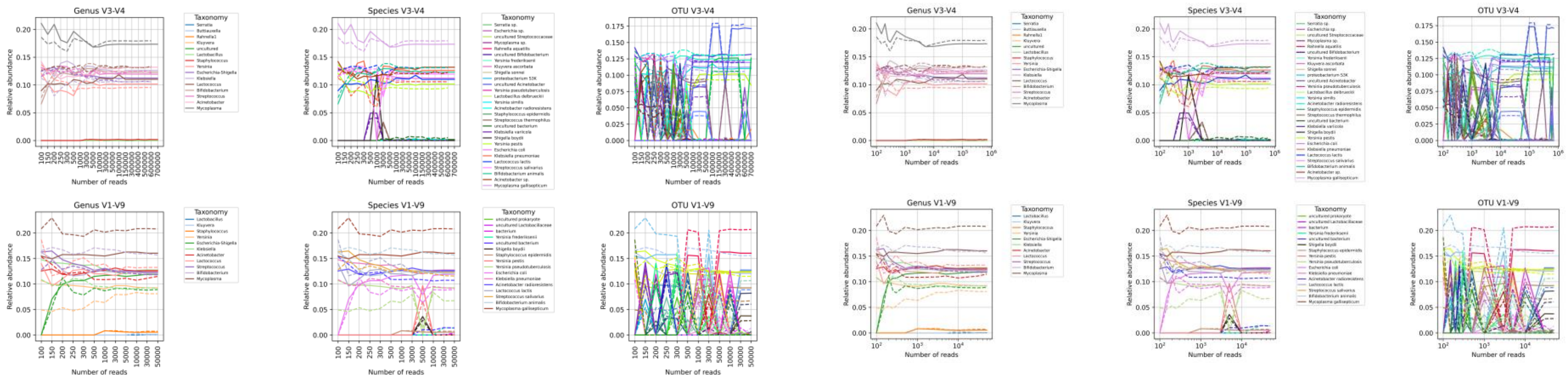

Supplementary 3 Fig.21 Change in relative representation as a function of the number of input reads (left - original, right - log scale). Bacterial community, **pool mode.**

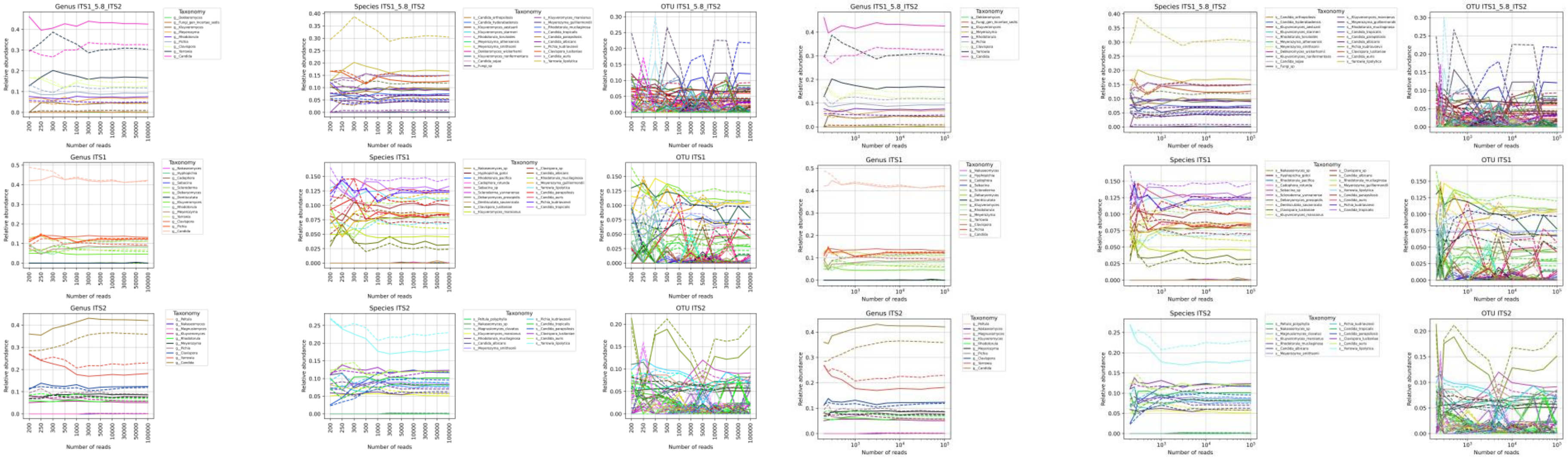

Supplementary 3 Fig.22 Change in relative representation as a function of the number of input reads (left - original, right - log scale). Fungal community, **pool mode.**

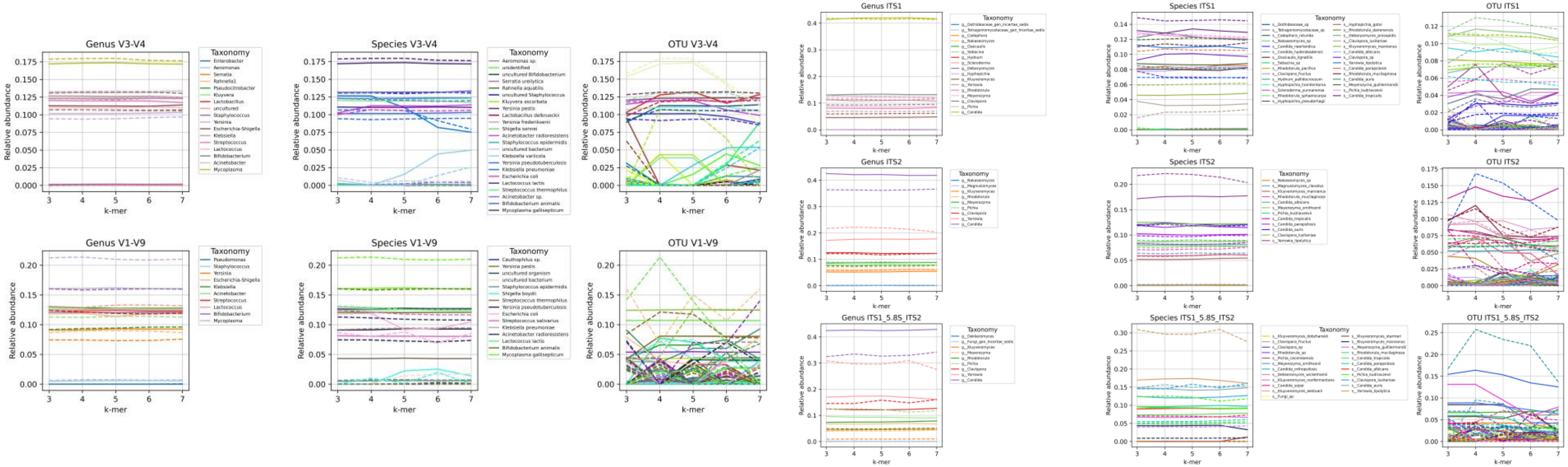

Supplementary 3 Fig.23 Change in relative representation as a different K-measure sizes (left–bacterial community, right –fungal community, **single mode**).

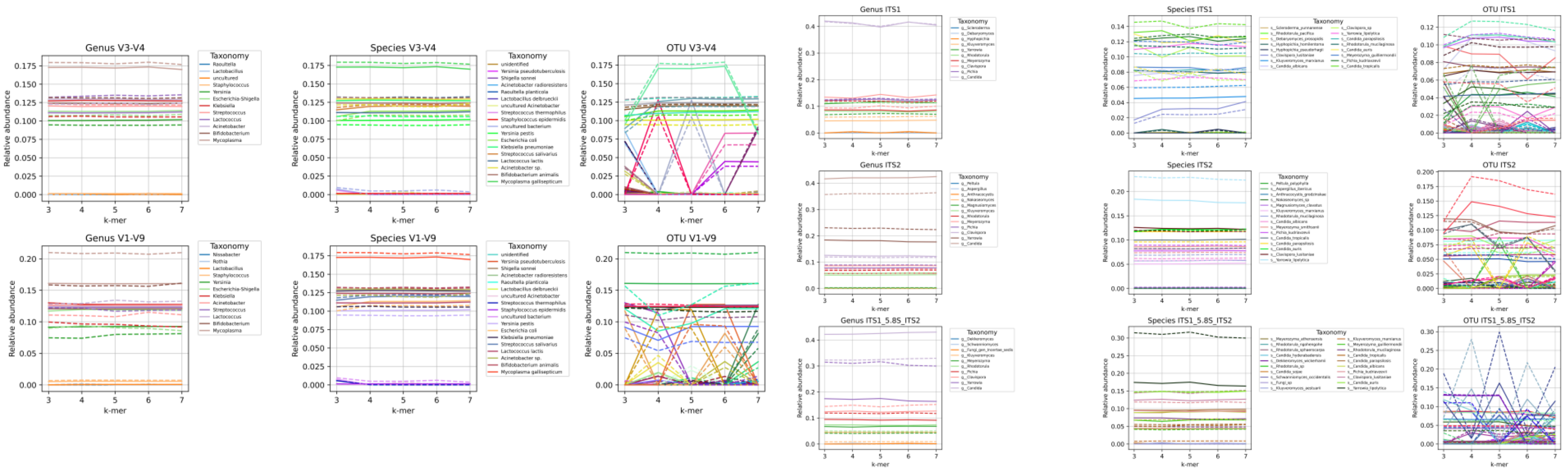

Supplementary 3 Fig.24 Change in relative representation as a different K-measure sizes (left–bacterial community, right –fungal community, **pool mode**).

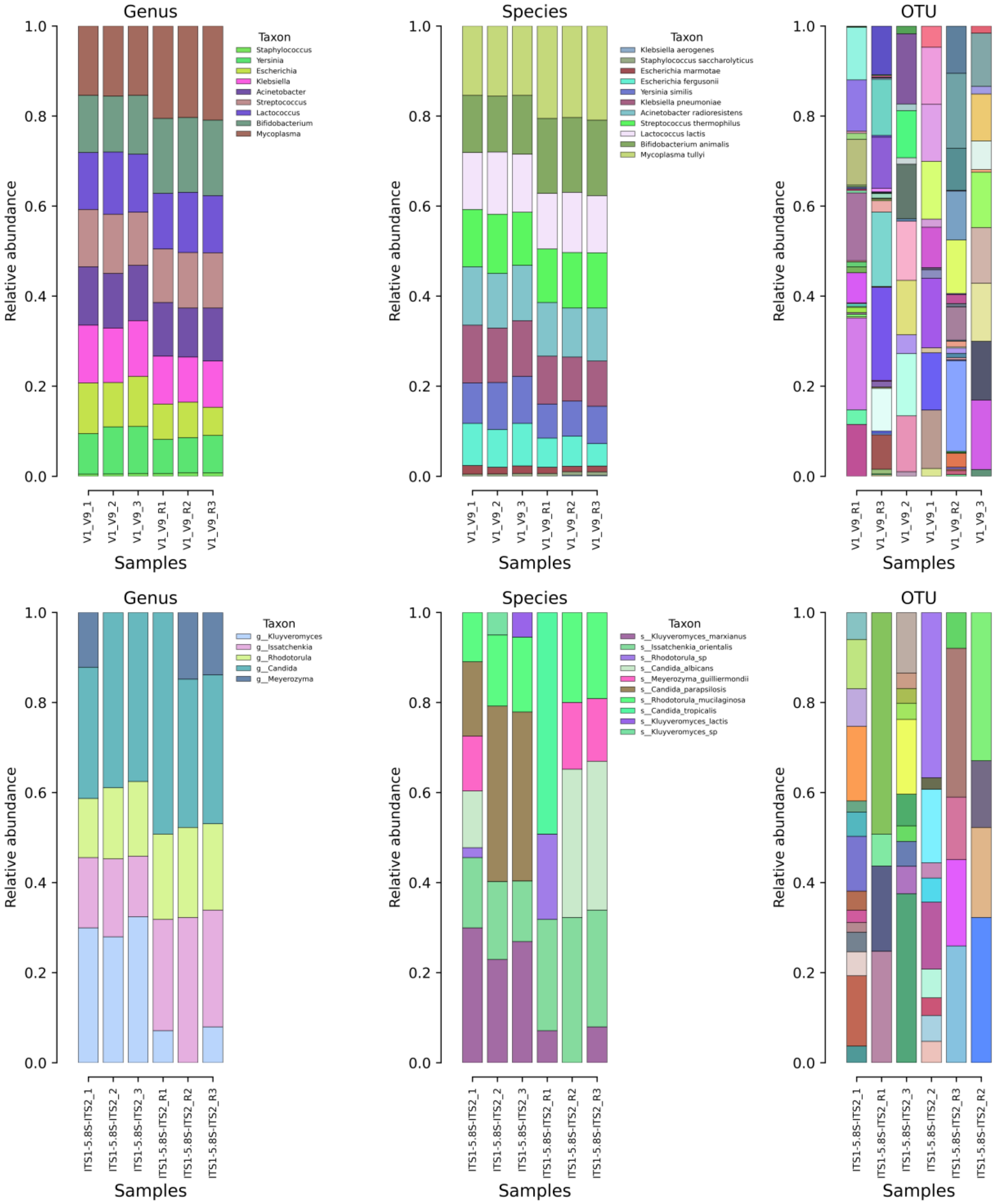

Supplementary 3 Fig.25 NanoCLUST results for the analysis of our mock communities.
